# Evolutionary divergence of photoprotection in the green algal lineage: a plant‐like violaxanthin de‐epoxidase enzyme activates the xanthophyll cycle in the green alga *Chlorella vulgaris* modulating photoprotection

**DOI:** 10.1111/nph.16674

**Published:** 2020-06-16

**Authors:** Laura Girolomoni, Francesco Bellamoli, Gabriel de la Cruz Valbuena, Federico Perozeni, Cosimo D'Andrea, Giulio Cerullo, Stefano Cazzaniga, Matteo Ballottari

**Affiliations:** ^1^ Department of Biotechnology University of Verona Strada le Grazie 15 Verona 37134 Italy; ^2^ IFN‐CNR Department of Physics Politecnico di Milano Piazza Leonardo da Vinci 32 Milan 20133 Italy; ^3^ Center for NanoScience and Technology @PoliMi Istituto Italiano di Tecnologia via Pascoli 70/3 Milan 20133 Italy

**Keywords:** carotenoids, *Chlorella vulgaris*, green algae, nonphotochemical quenching, photoprotection, photosynthesis, photosystem, xanthophyll cycle

## Abstract

- The xanthophyll cycle is the metabolic process by which the carotenoid violaxanthin is de‐epoxidated to zeaxanthin, a xanthophyll with a crucial photoprotective role in higher plants and mosses. The role of zeaxanthin is still unclear in green algae, and a peculiar violaxanthin de‐epoxidating enzyme was found in the model organism *Chlamydomonas reinhardtii*. Here, we investigated the molecular details and functions of the xanthophyll cycle in the case of *Chlorella vulgaris*, one of the green algae most considered for industrial cultivation, where resistance to high light stress is a prerequisite for sustainable biomass production.
- Identification of the violaxanthin de‐epoxidase enzyme in *C*.* vulgaris* was performed by genome mining and *in vitro* analysis of the catalytic activity of the gene product identified. The photoprotective role of zeaxanthin was then investigated *in vivo* and in isolated pigment‐binding complexes.
- The results obtained demonstrate the functioning, even though with a different pH sensitivity, of a plant‐like violaxanthin de‐epoxidase enzyme in *C*.* vulgaris*. Differently from *C*.* reinhardtii*, zeaxanthin accumulation in *C*.* vulgaris* was found to be crucial for photoprotective quenching of excitation energy harvested by both photosystem I and II.
- These findings demonstrate an evolutionary divergence of photoprotective mechanisms among Chlorophyta.

## Introduction

**Fig. 1:**
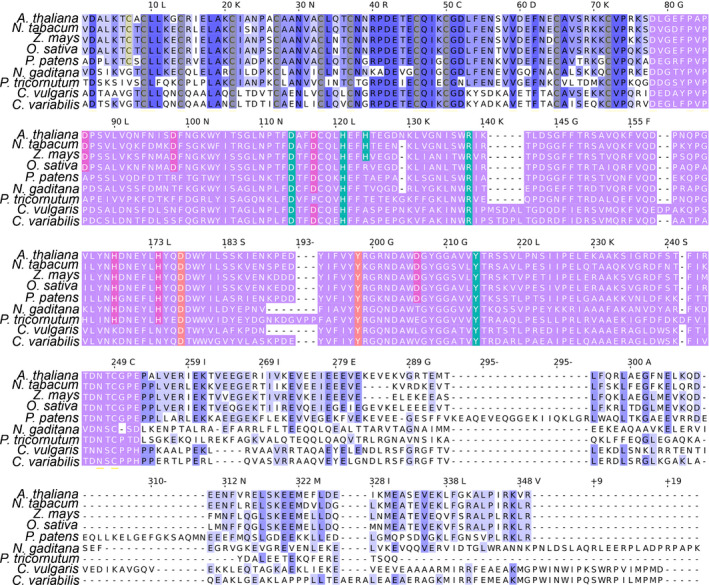
Multiple alignment of violaxanthin de‐epoxidase (VDE) enzyme sequences. Multiple alignment of VDE sequences retrieved from *Arabidopsis thaliana*, *Nicotiana tabacum*, *Zea mays*, *Oryza sativa*, *Physcomitrella patens*, *Nannochlopsis gaditana*, *Phaeodactylum tricornutum*, *Chlorella vulgaris*, and *Chlorella variabilis*. The domains organization is divided into three main regions: N‐terminus region (1–76 in *A*.* thaliana*) is the cysteine‐rich region, the central region (77–252, in violet color) is the lipocalin domain, and the C‐terminus part is the glutamic‐rich region. At the N‐terminus, the cysteine residues conserved are indicated in grey; the cysteines conserved only in land plants are reported in yellow. In the lipocalin domain, the residues important for the catalytic activity, for the structure organization, and for pH sensitivity are indicated, respectively, in orange, green, and purple.

Photosynthetic organisms use light energy to produce chemical energy by fixing CO_2_ into organic biomass. Light energy, absorbed by photosystems I (PSI) and II (PSII), is used to fuel the photochemical reactions by which electrons are transported from electron donors, as water, to NADP^+^. Light‐driven electron transport is coupled with proton transport from stroma to lumen, forming a transmembrane electrochemical gradient exploited by chloroplastic ATPase to produce ATP. NADPH and ATP are then used to fix CO_2_ into sugars by the Calvin–Benson cycle. The irradiance to which the photosynthetic machinery is exposed may undergo daily or seasonal changes; light may thus be limiting or in excess. In the latter case, the products of the photosynthetic light phase, ATP and NADPH, are not fully consumed by the Calvin–Benson cycle: the impaired regeneration of NADP^+^ and ADP by carbon fixation reactions causes a saturation of the photosynthetic electron transport, increasing the probability of excitation energy transfer from Chl triplets to oxygen (O) which forms the highly toxic reactive O species (ROS; Niyogi, 1999). Several acclimation responses have been observed at different timescales in photosynthetic organisms exposed to different light regimes. Long‐term acclimation responses involve changes at the level of pigments and pigment‐binding complexes accumulation, which are only partially conserved among the different species (Niyogi, 1999; Ballottari *et al*., 2007; Bonente *et al*., 2012; Allorent *et al*., 2013; Chaux *et al*., 2017). The main short‐term photoprotection mechanism activated in oxygenic photosynthetic organisms is nonphotochemical quenching (NPQ), by which Chl singlet excited states are dissipated into heat (Demmig‐Adams *et al*., 1996). NPQ has three different components, distinguishable by their kinetics. The fastest component activated upon illumination is the pH or energy‐dependent component, called qE (Horton *et al*., 1996; Müller *et al*., 2001). The mid‐range component is qT, related to the so‐called ‘state transitions’, a mechanism by which some antenna proteins of PSII, upon phosphorylation, move to PSI in order to balance the excitation pressure among the two photosystems (Wollman, 2001). Finally, the slowest component is related to the photoinhibition of photosynthesis by PSII degradation, a mechanism called qI (Horton *et al*., 1996), and/or to a zeaxanthin‐dependent slowly relaxing component called qZ (Nilkens *et al*., 2010). Other mid‐term adaptive responses involve chloroplasts movement to properly balance light absorption (Li *et al*., 2009) and sun tracking with specific movements of leaves (Greer & Thorpe, 2009). The role of zeaxanthin in NPQ has been long debated, with different reports supporting its direct (Holt *et al*., 2005; Ahn *et al*., 2008; Dall'Osto *et al*., 2017; Park *et al*., 2019) or indirect (Xu *et al*., 2015) contribution to quenching. Moreover, a possible role of zeaxanthin in PSI quenching has been reported in *Arabidopsis thaliana* (Ballottari *et al*., 2014) even if this quenching mechanism has been negatively argued by Tian *et al*. (2017). In higher plants, the xanthophyll cycle is triggered by luminal acidification and is mediated by the violaxanthin de‐epoxidase (VDE) enzyme, which converts violaxanthin into zeaxanthin in two de‐epoxidation steps, forming antheraxanthin as intermediate. Zeaxanthin is involved in singlet and triplet Chl excited states quenching and in ROS scavenging (Rockholm & Yamamoto, 1996; Betterle *et al*., 2010; Nilkens *et al*., 2010; Dall'Osto *et al*., 2012; Ballottari *et al*., 2014; Xu *et al*., 2015). VDE is a nuclear‐encoded protein activated by lumenal acidification, as a consequence of light phase saturation (Gilmore & Yamamoto, 1993), and requires ascorbate for its activity to reduce the epoxy group, with consequent water production (Arnoux *et al*., 2009). Previous studies revealed that the VDE activity is inhibited by dithiothreitol (DTT), which reduces one or more disulphide bonds formed by cysteine residues (Yamamoto & Kamite, 1972). The protein sequence of the *A*.* thaliana* VDE contains three main domains (Fig. 1): a cysteine‐rich region containing 11 cysteine residues conserved in different plant species (among which 10 are also conserved in algal species), a catalytic site, and a glutamate‐rich region (Simionato *et al*., 2015). Site‐directed mutagenesis experiments showed that, in the catalytic domain, the residues essential for the VDE activity are Asp177 and Tyr198, whereas the amino acids important for the structural organization are Asp114, Arg138, His121, and Tyr214 (Saga *et al*., 2010). Moreover, four histidine residues (His121, His124, His169, and His173 in the VDE sequence from spinach) have been reported to influence catalytic activity of VDE (Emanuelsson *et al*., 2003) and its pH‐dependent binding to the thylakoid membranes (Gisselsson *et al*., 2004). The pH‐dependent activity of VDE was also proved by substituting the protonatable residues with aliphatic amino acids (Fufezan *et al*., 2012). In microalgae, the role of the xanthophyll cycle does not seem to be homogeneous: zeaxanthin accumulation in the model green alga *Chlamydomonas reinhardtii* has been reported to be important for ROS scavenging, but its role in NPQ induction, if any, is minor (Niyogi *et al*., 1997; Bonente *et al*., 2011; Quaas *et al*., 2015). Differently, a partial de‐epoxidized xanthophylls‐dependent NPQ has been reported in some species belonging to green algae (Quaas *et al*., 2015), brown algae (García‐Mendoza & Colombo‐Pallotta, 2007), diatoms (Lavaud *et al*., 2012), eustigmatophytes (Chukhutsina *et al*., 2017), and Alveolata (Kaňa *et al*., 2016). In the diatoms *Phaeodactylum tricornutum* and *Thalassiosira pseudonana*, genes encoding for a plant‐like VDE have been reported to be involved in de‐epoxidation of the xanthophyll diadinoxanthin producing diatoxanthin, with this peculiar xanthophyll cycle being involved in NPQ induction (Lavaud *et al*., 2012). In these photosynthetic heterokonts, two additional VDE‐like (VDL) or VDE‐related (VDR) enzymes have been reported, even if their catalytic activity and their physiological role are still under debate (Bertrand, 2010). In the case of *C*.* reinhardtii*, the catalytic violaxanthin de‐epoxidation activity has been recently attributed to an enzyme, called chlorophycean VDE (CVDE), which is not related to the plant‐VDE but to a lycopene cyclase from photosynthetic bacteria (Li *et al*., 2016). A similar CVDE enzyme has been more recently reported also in case of other green algae, such as *Volvox carteri* and *Chromochloris zofingiensis* (Roth *et al*., 2017). This observation led to the hypothesis that green algae and plants evolved different VDE enzymes, with implications for their regulation and functions (Li *et al*., 2016).

In this work, we investigate, both *in vivo* and *in vitro*, the molecular details of the enzyme responsible for zeaxanthin accumulation in one of the most promising green algae for industrial cultivation: *Chlorella vulgaris* (Lowrey *et al*., 2015; Zuniga *et al*., 2016; Sarayloo *et al*., 2017; Guarnieri *et al*., 2018; Cecchin *et al*., 2019). It has been recently reported that understanding and control of the molecular mechanisms involved in high light resistance is an important biotechnological tool in order to improve biomass productivity in microalgae (Treves *et al*., 2013; Treves *et al*., 2016), and in particular in *C*.* vulgaris* (Dall'Osto *et al*., 2019). We demonstrate that *C*.* vulgaris* undergoes a plant‐like xanthophyll cycle and that zeaxanthin is involved in quenching excitation energy absorbed by both PSI and PSII. Our results demonstrate a divergence in molecular mechanism and function of the xanthophyll cycle among green algae, probably associated with the varying evolutionary pressures to which the different species were exposed in their specific habitats. These results pave the way for a better understanding of the evolution of photoprotective mechanisms in photosynthetic organisms, to improve photosynthetic efficiency by properly tuning energy dissipative pathways (Kromdijk *et al*., 2016).

## Materials and Methods

### Strains and culture conditions

*Chlorella vulgaris* (CCAP211/11P) and *C*.* reinhardtii* (4A+) cells were grown at 25°C in flasks on a shaker at 160 rpm with a white light at 60 µmol m^−2^ s^−1^ with a 16 h : 8 h, light : dark photoperiod in BG‐11 medium (Allen & Stanier, 1968) or HS medium (Kropat *et al*., 2011), respectively.

### *Arabidopsis thaliana* genotypes and growth conditions

*Arabidopsis thaliana* Columbia‐0 (Col‐0) ecotype WT and *npq1* plants (Niyogi *et al*., 1998) were grown under controlled conditions at an irradiance of 300 μmol m^−2^ s^−1^ with a 16 h : 8 h, photoperiod, temperature of 23°C : 20°C, day : night, and 50–70% relative air humidity.

### Violaxanthin de‐epoxidase identification and phylogenetic analysis

Putative VDE genes were searched in the assembled *C*.* vulgaris* genome by Blast search using *A*.* thaliana* VDE1 (AT1G08550) as query and *C*.* vulgaris* translated genome as database. All the sequences used for phylogenetic analysis were retrieved from Uniprot. Sequences carrying a VDE lipocalin domain were retrieved from InterPro (IPR010788). Sequence alignment was obtained by Maffd (v.7.394) software. The phylogenetic trees were generated using ClustalOmega with default parameters, trimAL for alignment cleaning and Phyml with 100 bootstraps, and rendered with Ete 3 toolkit (Huerta‐Cepas *et al*., 2016).

### Violaxanthin de‐epoxidase expression and purification

Total RNA from *C*.* vulgaris* was extracted from cells grown in high light using the Direct‐zol™ RNA Miniprep Plus kit (Zymo Research, Irvine, CA, USA). Transcript sequence was amplified from complementary DNA using specific primers designed on transcript g7391 (Supporting Information Methods S1). Mature VDE coding sequence was cloned into pET28 expression vector not including the initial 28 amino acids putative signal peptide for the chloroplast. The signal peptide was calculated using ChloroP 1.1 and TargetP 2.0 tools, considering the shortest result obtained. Potential additional residues involved in protein import in thylakoid lumen identified by TargetP are reported in Methods S1; these residues were anyway maintained in the recombinant *C*.* vulgaris* VDE protein produced, since they are not expected to influence protein activity. His‐tag was added at the C‐terminus. The pQE60 construct for expression of *A*.* thaliana* VDE enzyme in *Escherichia coli*, previously described (Hieber *et al*., 2002; Fufezan *et al*., 2012), was kindly gifted by Professor Tomas Morosinotto (University of Padua, Italy). Recombinant *A*.* thaliana* and *C*.* vulgaris* VDE were expressed in *E*.* coli* Origami™ 2(DE3) ( Merck KGaA, Darmstadt, Germany) by inducing cells with 1 mM isopropyl β‐d‐1‐thiogalactopyranoside for 5 h at 37°C and purified by nickel affinity column as described in Saga *et al*. (2010).

### Pigment analysis

Pigments were extracted from whole cells using dimethyl sulphoxide (DMSO) and from thylakoids and purified pigments‐binding complexes with 80% acetone. In the case of the pigments mixture used for VDE activity assays, carotenoids were extracted with diethyl ether inducing phase separation: the organic phase was collected, dried in a SpeedVac, and resuspended in 80% acetone for high‐performance liquid chromatography (HPLC) analysis (Saga *et al*., 2010). DMSO or acetone extracts were then centrifuged and the supernatants analysed by HPLC as described by Lagarde *et al*. (2000) using a Jasco LC‐4000 (Jasco Europe SRL, Cremella LC, Italy) instrument equipped with Synergy hydro‐rp 80 (Phenomenex, Torrance, CA, USA) C18 column. Pigments were separated by a 15 min gradient of ethyl acetate (0–100%) in acetonitrile–water–triethylamine (9 : 1 : 0.01, v/v) at a flow rate of 1.5 ml min^−1^. The de‐epoxidation index (DI) was calculated from the concentration of zeaxanthin, antheraxanthin, and violaxanthin as:

$\text{DI}=\frac{\left[\text{Zeaxanthin}]+0.5[\text{Antheraxanthin}\right]}{\left[\text{Zeaxanthin}]+[\text{Antheraxanthin}]+[\text{Violaxanthin}\right]}$

### Violaxanthin de‐epoxidase activity assay

VDE activity was tested by adding pure violaxanthin as substrate in a de‐epoxidation buffer as described by Saga *et al*. (2010). In particular, the de‐epoxidation buffer was composed of 67 mM citrate buffer at pH 5.1, 60 mM ascorbate, 6% methanol, 0.33 μM violaxanthin, and 9 µM monogalactosyldiacylglycerol (MGDG). Violaxanthin and MGDG were mixed in methanol and then added to the de‐epoxidation buffer. The inhibitor DTT was added to the de‐epoxidation buffer at a concentration of 1 mM when indicated in the text. Violaxanthin de‐epoxidation was monitored by measuring changes in absorption spectra in the 480–520 nm spectral region (Saga *et al*., 2010) and by HPLC analysis (Lagarde *et al*., 2000).

### *In vitro* de‐epoxidation on thylakoids

*Chlorella vulgaris* and *C*.* reinhardtii* thylakoids were extracted from overnight dark‐adapted cells by destroying cells with glass beads directly in the de‐epoxidation buffer (40 mM MES pH 5.1, 330 mM sorbitol, 5 mM magnesium chloride (MgCl_2_), 10 mM sodium chloride (NaCl), 20 mM ascorbate, and 0.5% BSA). The inhibitor DTT was added to the de‐epoxidation buffer at a concentration of 1 mM when indicated in the text. In the case of spinach, leaves were ground in 0.4 M NaCl, 5 mM MgCl_2_, 20 mM tricine/potassium hydroxide pH 7.8 and 0.5% BSA, filtered through a 10 µm filter, centrifuged at 10 000 ***g*** and then resuspended in the de‐epoxidation buffer. The de‐epoxidation reaction was then performed at 20°C up to 1 h in the case of spinach and up to 8 h in the case of *C*.* vulgaris* and *C*.* reinhardtii*. Pigments were then extracted using 80% acetone and analysed by HPLC.

### Sodium dodecyl sulphate polyacrylamide gel electrophoresis and Western blotting

Total protein extracts were loaded into sodium dodecyl sulphate polyacrylamide gel electrophoresis (PAGE) 12% gels as described by Laemmli (1970). Western blot analysis was performed using antibody for *A*.* thaliana* VDE (Ballottari *et al*., 2007).

### Nonphotochemical quenching measurements

NPQ was calculated as $\left(F_{m}-F_{m}^{\prime }\right)/F_{m}^{\prime }$ (Bilger & Björkman, 1990) using a Dual PAM‐101 (Waltz, Effeltrich, Germany). *F*_m_ is the maximum Chl fluorescence emitted by dark‐adapted cells after 2 min treatment with a far‐red LED, $F_{m}^{\prime }$ is the maximum fluorescence measured upon exposure to actinic light or during dark recovery. Far‐red light was turned on also during dark recovery. A 5000 µmol m^−2^ s^−1^ saturation pulse was used to induce *F*_m_ and $F_{m}^{\prime }$ while the intensity of the actinic lights used were reported in the results section.

### 77 K quenching analysis

The 77 K fluorescence emission was recorded as described by Girolomoni *et al*. (2019) on whole *C*.* vulgaris* cells dark adapted or high light treated (2000 μmol m^−2^ s^−1^). DTT was added as described in the text to inhibit VDE catalytic activity. Green fluorescent protein (GFP) was added to the samples as an internal standard for normalization of fluorescence emission spectra.

### Thylakoid solubilization and pigments‐binding complexes purification

Pigment binding complexes were separated by Deriphat‐PAGE as described previously (Dreyfuss & Thornber, 1994). Deriphat‐PAGE gel was loaded with thylakoid membranes solubilized in 0.8% *n*‐dodecyl‐β‐d‐maltoside (Cazzaniga *et al*., 2014). Trimeric light‐harvesting complex (LHC) II was also isolated by thylakoid membranes solubilization in 0.6% *n*‐dodecyl‐α‐d‐maltoside and ultracentrifugation in a sucrose gradient as described previously (Caffarri *et al*., 2001).

### Time‐resolved fluorescence

Time‐resolved fluorescence measurements were performed by time‐correlated single photon counting using a ChronosBH ISS Photon Counting instrument (ISS, Inc., Champaign, IL, USA) with picosecond laser excitation at 447 nm operating at 50 MHz. Laser power was kept below 0.1 μW. Fluorescence emission was acquired at 690 nm with a bandwidth of 4 nm. In the case of PSI complexes, time‐resolved fluorescence decay measurements were carried out using a femtosecond excitation laser (Chameleon Ultra II, Coherent, Santa Clara, CA, USA) at 440 nm operating at 80 MHz and a streak camera detection system (C5680, Hamamatsu Photonics Italia SRL, Milan, Italy), as reported by Ballottari *et al*. (2014). Fluorescence decay kinetics were obtained after integration over the whole emission spectrum and then fitted with exponential functions.

### Data availability

The data and materials described fully herein are available from the corresponding author upon reasonable request.

## Results

### Identification of violaxanthin de‐epoxidase enzyme in *Chlorella vulgaris*

*Chlorella vulgaris* genomic and transcriptomic data were used to mine possible VDE and CVDE genes. Local Blast analysis on the *C*.* vulgaris* genome (Cecchin *et al*., 2019), performed using the *A*.* thaliana* VDE sequence as query, gave a positive result in the case of gene *g7391*, whereas the same analysis performed using the *C*.* reinhardtii* CVDE enzyme identified *g3843* as putative homologue for CVDE in *C*.* vulgaris*. Whereas in the case of CVDE there is limited information available regarding the key residues involved in its catalytic function, the structure of the catalytic domain and the function of specific residues have been previously reported for VDE (Emanuelsson *et al*., 2003; Gisselsson *et al*., 2004; Arnoux *et al*., 2009; Saga *et al*., 2010; Fufezan *et al*., 2012; Simionato *et al*., 2015). The putative *C*.* vulgaris* VDE protein sequence was thus compared with VDE sequences from higher plants or other microalgae species. Multiple alignment (Fig. 1) shows high similarity of *C*.* vulgaris* VDE with the other VDE sequences analysed in the case of the catalytic domain. The N‐terminal domain of *C*.* vulgaris* VDE is enriched in cysteine residues, a conserved feature compared with VDE sequences from all the organisms analysed. The multiple alignment also reveals the conservation in *C*.* vulgaris* of the key residues for catalytic activity (Asp177 and Tyr198) previously reported in the case of higher plants (Saga *et al*., 2010). Important residues for the structural organization, Asp114, His121, Arg138 and Tyr214, are conserved in all the VDE sequences reported in Fig. 1, whereas some variations can be observed in the case of the other His residues, His124, His169 and His174 (corresponding in *A*.* thaliana* to the His168 and His173 previously investigated in VDE from spinach). These residues are reported to influence the catalytic activity (Emanuelsson *et al*., 2003) and pH‐dependent binding of VDE to the thylakoid membranes (Gisselsson *et al*., 2004): His124 is present only in higher plants, with the exclusion of *Oryza sativa*; His168 and His174 are conserved in higher plants, mosses, and diatoms but not in the green algae *C*.* vulgaris* and *Chlorella variabilis*, where they are substituted with the basic residues lysine and asparagine, respectively. Considering the protonatable residues involved in pH sensing, other variations are evident: Asp 114 is conserved in all the sequences analysed but not in *Phaeodactylum tricornutum*, Asp96 and Asp98 are conserved only in VDE sequence from higher plants, and Asp206 is conserved only in land plants (Fig. 1). These results open the question about a possible different pH dependence of *C*.* vulgaris* VDE enzyme compared with VDE enzymes from higher plants.

### Phylogenetic distribution of violaxanthin de‐epoxidase and chlorophycean violaxanthin de‐epoxidase

**Fig. 2:**
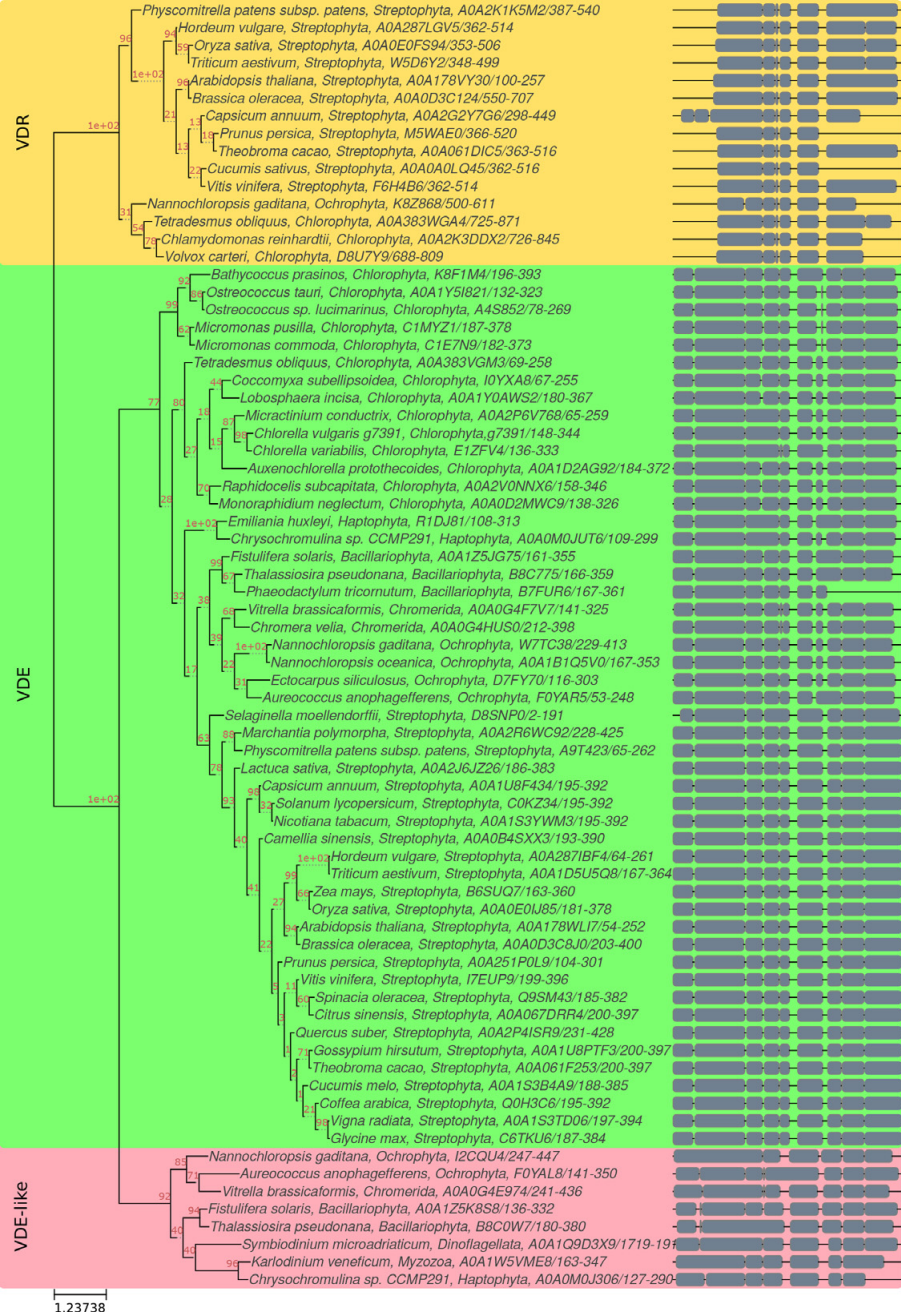
Phylogenetic tree of violaxanthin de‐epoxidase (VDE), VDE‐related (VDR) and VDE‐like proteins. The phylogenetic tree was obtained by multiple alignment of protein sequences carrying a VDE lipocalin domain identified by Interpro (IPR010788). The units of branch length are residues substitution per site divided by the length of the sequence. Bootstrap values are reported in red.

To investigate the distribution of VDE among different photosynthetic organisms, a phylogenetic tree of putative VDE enzymes was then assembled. Protein sequences with the VDE lipocalin domain identified by InterProScan were used to assemble a phylogenetic tree with the C.* vulgaris* VDE. As reported in Fig. 2, VDE enzymes from Streptophyta clustered together, whereas a different cluster could be identified containing VDE enzymes from some green algae (among which are *C*.* vulgaris*, *C*.* variabilis*, *Auxenochlorella prototechoides*, *Monoraphydium neglectum*, *Lobopshera incisa* and *Ostreococcus tauri*), and from organisms whose plastids originated by a secondary symbiosis (such as diatoms, Haptophyta, Ochrophyta, and photosynthetic Alveolata such as *Chromera velia*). Separate and more divergent groups at the two ends of the phylogenetic tree, including VDL enzymes, were also identified: a group of VDR enzymes from organisms whose plastids originated by a secondary symbiosis, and at the opposite end a cluster including VDL enzymes from Streptophyta and Chlorophyta, with no VDE function reported yet. In order to investigate the presence of VDE or CVDE enzymes in Chlorophyta, a more detailed analysis was performed among these organisms. The phylogenetic distribution of CVDE and its homologue CruP (Li *et al*., 2016) among green algae is reported in Fig. S1, showing separate clusters for CVDE and CruP. The identification of VDE, VDR and CVDE genes among green algae species with complete genomes available is reported in Table S1: no VDE enzyme could be found in the Volvocales species analysed herein, such as *C*.* reinhardtii*, *Chlamydomonas eustigma*, *Gonium pectorale*, and *Volvox carteri*, where CVDE enzymes could be found instead. Differently, in the case of the Mamiellales species, only VDE enzymes could be found and no CVDE. Sphaeropleales species *O*.* tauri*, *Ostreococcus lucimarinus*, *Micromonas commoda* and *Micromonas pusilla* were instead characterized for having both VDE and CVDE enzymes. A more variable situation could be observed in the case of Trebouxiophyceae: all the species analysed contain a VDE gene, with the exception of *Chlorella sorokiniana*, where a VDR gene can be found, whereas CVDE genes could be identified only in the case of *Auxenochlorella protothecoides*, *C*.* sorokiniana*, *Coccomyxa subellipsoidea* and *C*.* vulgaris*. Even if further experimental work is required to investigate the violaxanthin de‐epoxidation reactions in the different species reported herein, these results indicate a divergency among Chlorophyta of violaxanthin de‐epoxidation catalysis during evolution.

### *In vitro* and *in vivo* de‐epoxidation

**Fig. 3:**
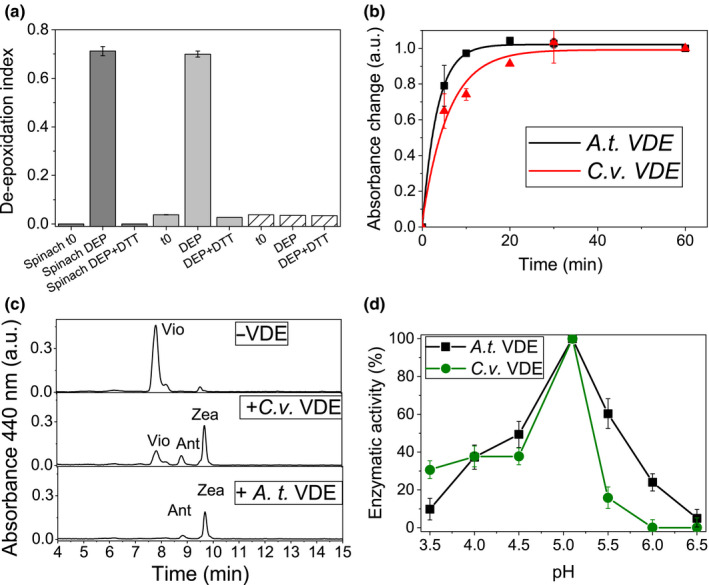
*In vitro* violaxanthin de‐epoxidation assay. (a) De‐epoxidation index of thylakoids isolated from spinach, *Chlorella vulgaris* (*C*.*v*.) and* Chlamydomonas reinhardtii* (*C*.*r*.) before (t0) or after (DEP) 1 h (spinach) or 8 h (*C*.*v*. and *C*.*r*.) at pH 5.1 in the presence of ascorbate in order to induce violaxanthin de‐epoxidation. De‐epoxidation index obtained in presence of violaxanthin de‐epoxidase (VDE) inhibitor dithiothreitol (DTT; 1 mM) is also reported. (b) VDE activity of recombinant *C*.* vulgaris* evaluated *in vitro* by measuring pigments absorption changes at 505 nm due to violaxanthin conversion to zeaxanthin. The absorption at 540 nm was subtracted from the absorption at 505 nm; absorption kinetics were normalized to 1 for the maximum activity observed. (c) Chromatogram related to high‐performance liquid chromatography pigment analysis after de‐epoxidation *in vitro* assay in presence or absence of VDE enzymes from *C*.* vulgaris* or *Araidopsis thaliana* (*A*.*t*.) measured after 60 min of incubation. (d) VDE enzymatic activity during *in vitro* enzymatic assay at different pH, normalized to 100% in the case of the maximum value (pH 5.1). SDs are reported as error bars (*n* = 3). Ant, antheraxanthin; Vio, violaxanthin; Zea, zeaxanthin.

The role of VDE and/or CVDE enzymes in the xanthophyll cycle activation was investigated in *C*.* vulgaris* both *in vitro* and *in vivo*. Differently from CVDE proteins, VDE enzymes from higher plants have been reported *in vitro* to catalyse violaxanthin de‐epoxidation in thylakoids in the presence of ascorbate and low pH, and DTT inhibits their activity (Adams *et al*., 1990). *Chlorella vulgaris*, spinach, and *C*.* reinhardtii* thylakoids were exposed at pH 5.1 in the presence of 20 mM ascorbate as reducing agent to activate the VDE enzyme in the presence or absence of 1 mM DTT. As reported in Fig. S2, substantial violaxanthin de‐epoxidation was detectable already after 30 min in the case of spinach, whereas 4–8 h were required to induce zeaxanthin formation in *C*.* vulgaris*. In both cases, a specific inhibitory activity of DTT was evident, causing no violaxanthin de‐epoxidation (Figs 3a, S3). By contrast, in the case of *C*.* reinhardtii*, no violaxanthin de‐epoxidation was observed in experimental conditions, in agreement with previous observations (Li *et al*., 2016). Considering the inactivity of CVDE *in vitro*, the potential activity of *C*.* vulgaris* CVDE was then investigated *in vivo*.* Chlorella vulgaris* cells were exposed to light of 2000 μmol m^−2^ s^−1^ for up to 40 min in the presence or absence of the inhibitor DTT (1 mM). As reported in Fig. S4, the DI increased upon light exposure in the absence of DTT, whereas cells treated with this inhibitor showed an almost completely impaired zeaxanthin accumulation, consistent with previous findings in this species (Goss *et al*., 2006; Quaas *et al*., 2015). This result suggests a minor role of the DTT‐insensitive CVDE xanthophyll cycle activation in *C*.* vulgaris*. In order to investigate the catalytic activity of the *C*.* vulgaris* VDE enzyme identified, its coding sequence was cloned in expression vector and overexpressed in *E*.* coli* as previously reported (Saga *et al*., 2010). Recombinant protein was then purified from the soluble fraction of lysate bacterial cells through an affinity column and used for evaluating its catalytic activity in the presence of violaxanthin. An *in vitro* enzymatic assay was performed at pH 5.1 in the presence of violaxanthin and ascorbate by following the changes in the absorption spectrum (Fig. 3b): in the case of violaxanthin de‐epoxidation, an increase of the 500 nm absorption is expected due to zeaxanthin formation (Saga *et al*., 2010). *Arabidopsis thaliana* VDE was used as a positive control (Saga *et al*., 2010). An increase of the 505 nm absorption was indeed observed in the presence of recombinant VDEs from both *A*.* thaliana* and *C*.* vulgaris*, but not in the negative control (no VDE added), suggesting violaxanthin de‐epoxidation. Zeaxanthin accumulation in *A*.* thaliana* or *C*.* vulgaris* was then confirmed by HPLC analysis (Fig. 3c): after 60 min of incubation, the efficiency of violaxanthin de‐epoxidation was 95% and 77% for the *A*.* thaliana* and *C*.* vulgaris* VDE enzymes, respectively. The pH dependence of *C*.* vulgaris* VDE was then investigated by repeating the enzymatic *in vitro* assay at different pH after 60 min of incubation (Fig. 3d). While for both the *A*.* thaliana* and *C*.* vulgaris* VDE enzymes the optimum pH for the de‐epoxidation reaction was 5.1, the *C*.* vulgaris* subunit exhibited a reduced activity at higher pH compared with *A*.* thaliana* VDE. Considering the reduced content of protonatable residues involved in pH sensing in *C*.* vulgaris* VDE compared with *A*.* thaliana* VDE, the reduced activity of the former at higher pH suggests a possible cooperative regulation of pH sensing in this enzyme, partially affected in the *C*.* vulgaris* subunit (Fig. 1).

### Role of zeaxanthin in nonphotochemical quenching induction in *Chlorella vulgaris*

**Fig. 4:**
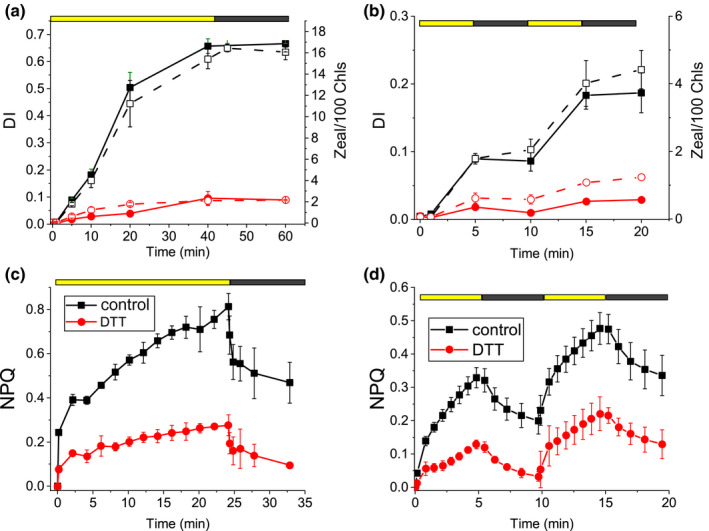
Violaxanthin de‐epoxidase (VDE) inhibition by dithiothreitol (DTT) and role of zeaxanthin in nonphotochemical quenching (NPQ) induction. DTT inhibitor (1 mM) was added to whole cells in order to inhibit VDE catalytic activity. Samples treated with DTT are reported in red circles, and samples where DTT was not added are reported in black squares. (a, b) De‐epoxidation index (DI; closed symbols) and zeaxanthin (Zea) content normalized to 100 Chls (open symbols) of cells treated in presence or absence of DTT for 40 min with irradiance of 2000 μmol m^−2^ s^−1^ followed with (a) 20 min of dark recovery or (b) illuminated for two consecutive cycles of 5 min light followed by 5 min of dark recovery. (c, d) Effect of DTT on NPQ kinetics: NPQ induction kinetics in presence or absence of DTT upon illumination with 2000 μmol m^−2^ s^−1^ actinic light for (c) 25 min followed by 10 min of dark recovery or (d) upon two consecutive cycles of 5 min light followed by 5 min dark. SDs are reported as error bars (*n* = 3).

The specific role of zeaxanthin in NPQ induction was then studied by measuring NPQ in the presence or absence of DTT, in order to inhibit VDE activity, under continuous light for 25 min, followed by dark recovery (Fig. 4a), or upon a double cycle of illumination interrupted by 5 min of dark. In this way, zeaxanthin accumulation is induced in the first cycle and its potential role in NPQ can be highlighted in the second cycle due to the long time required for zeaxanthin epoxidation (Fig. 4b). As reported in Fig. 4, the presence of DTT caused a strong decrease of NPQ during continuous light exposure or during both the first and second cycles of actinic light illumination, suggesting a key role of zeaxanthin for NPQ induction. Potential side effects of DTT can be excluded at the concentration used (1 mM), as evidenced by the similar maximum quantum yield (*F*_v_/*F*_m_) of PSII in *C*.* vulgaris* kept in the dark in the presence or absence of DTT for up to 30 min (Fig. S4), consistent with previous reports on isolated chloroplasts (Neubauer, 1993). By contrast, in the case of *C*.* reinhardtii*, the NPQ traces during the first and the second cycles were similar and no effect of DTT was evident (Fig. S5), confirming the minor role of zeaxanthin in this organism and the absence of CVDE inhibition by DTT (Li *et al*., 2016).

The correlation between zeaxanthin accumulation and NPQ induction kinetics in *C*.* vulgaris* was then investigated at different actinic light intensities ranging from 200 to 2500 μmol m^−2^ s^−1^ (Fig. S6). As shown in Fig. S7, an increase of the light intensity caused an increase of DI up to 0.6 at the highest irradiances. Similarly, higher actinic light intensities caused an increase of the maximum amplitude of NPQ induction, with reduced recovery in the dark (Fig. S7). The possible relation between NPQ induction and zeaxanthin accumulation was thus investigated by plotting the NPQ values measured at the end of the actinic light treatment and its components qE and qI (or qZ) as a function of the measured DI or zeaxanthin content (Fig. S7): an exponential asymptotic correlation could be drawn between DI and NPQ, qE, or qI (qZ). Interestingly, in the case of qE, the rate of the exponential asymptotic correlation function was higher than for NPQ and qI/qZ, with the latter having the lowest rate. These results demonstrate that xanthophyll cycle activation is almost linearly correlated with the slowly relaxing component of NPQ, whereas relatively few molecules of zeaxanthin are correlated with the activation of qE (Fig. 4).

### Role of zeaxanthin in photosystem I quenching in *Chlorella vulgaris*

**Fig. 5:**
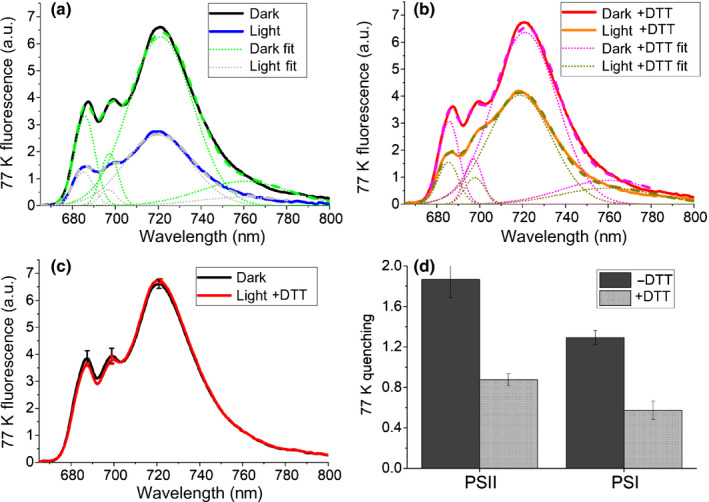
Photosystem (PS)I and PSII quenching measured at 77 K. (a, b) Fluorescence spectra were recorded for *Chlorella vulgaris* whole cells dark adapted (black/red) or high light treated (2000 μmol m^−2^ s^−1^) for 6 min (blue/orange) in (a) absence or (b) presence of violaxanthin de‐epoxidase inhibitor dithiothreitol (DTT; 1 mM). Green fluorescent protein was added as internal standard for normalization. Fluorescence spectra were reconstructed by spectral deconvolution with Gaussians: different Gaussians used are reported in green (dark‐adapted samples, no DTT), pink (dark‐adapted samples with DTT), grey (high‐light‐treated samples, no DTT), or dark yellow (high‐light‐treated samples with DTT). Peaks of the different Gaussians are reported. (c) Comparison of dark‐adapted samples in presence or absence of DTT, showing no major side effect due to the presence of the inhibitor. (d) Calculations of PSII and PSI quenching from the sum of the areas of the Gaussians used for the fitting according to the formula (*A*_dark_ − *A*_light_)/*A*_light_, where *A*_light_ and *A*_dark_ are, respectively, the amplitude after light treatment or at time zero of the different Gaussians attributable to PSII (peaking at 686 and 698 nm) or PSI (peaking at 720 nm). SDs are reported as error bars (*n* = 4).

NPQ analysis at room temperature only allows assessment of quenching events at the level of PSII, since the PSI fluorescence is almost undetectable. In order to investigate a possible role of zeaxanthin in PSI photoprotection, emission of whole cells was investigated at 77 K, where photochemical reactions are blocked and PSI fluorescence can be measured (Girolomoni *et al*., 2019). As reported in Fig. 5, by using GFP as an internal standard, a light‐dependent decrease of both PSII (680–695 nm) and PSI (720 nm) emission peaks was evident, as previously reported in the case of *C*.* reinhardtii* (Girolomoni *et al*., 2019). In the presence of DTT, inhibiting zeaxanthin accumulation, a less evident quenching of both PSII and PSI peaks was measured (Fig. 5). Gaussian deconvolution of emission spectra allowed us to retrieve the contributions of PSII and PSI to the 77 K emission spectra and to calculate the extent of light‐dependent quenching specifically for PSI and PSII. Consistently with NPQ measured at room temperature (Fig. 4), PSII quenching was strongly reduced in the presence of DTT. Similarly, PSI quenching was also reduced when VDE was inhibited, suggesting a role of zeaxanthin in PSI photoprotection.

### Distribution and quenching properties of zeaxanthin in pigment‐binding complexes

**Fig. 6:**
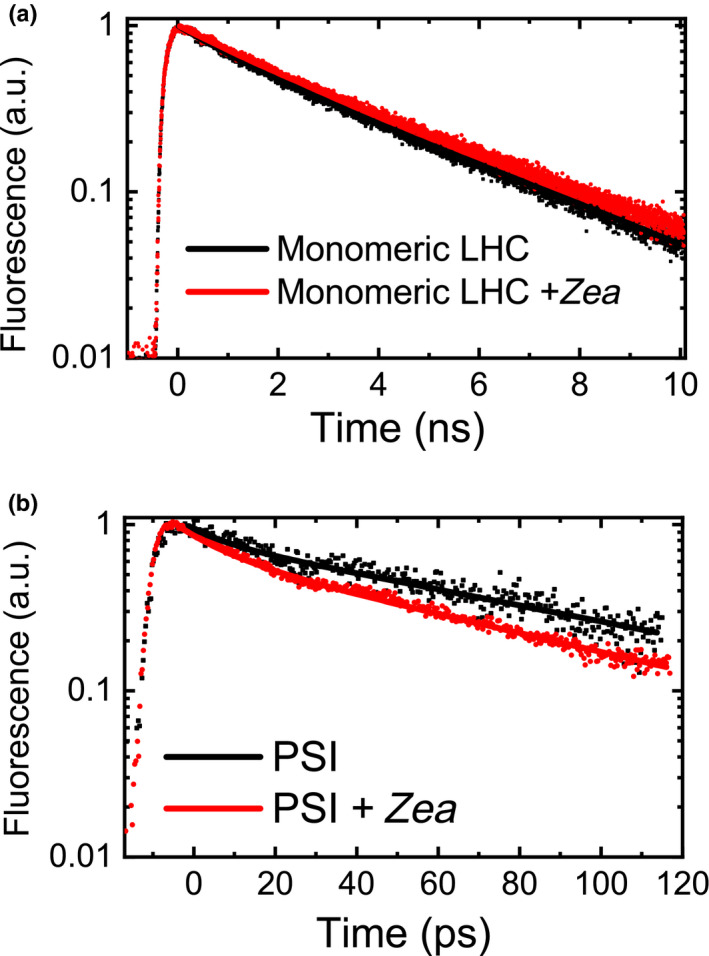
Fluorescence decay kinetics of isolated monomeric light‐harvesting complex (LHC) and photosystem I (PSI) complexes with and without zeaxanthin (*Zea*). Monomeric LHC and PSI complexes were isolated from *Chlorella vulgaris* thylakoid membranes before (black) or after (red) *in vitro* de‐epoxidation to induce zeaxanthin accumulation (bands A1/B1 and A8/B8 in Deriphat polyacrylamide gel electrophoresis gel reported in Supporting Information Fig. S8). Fluorescence decay kinetics were measured by (a) time‐correlated single photon counting in the 0–10 ns timescale for monomeric LHC and by (b) streak camera charge‐coupled device in the 0–200 ps timescale for PSI complexes.

Zeaxanthin distribution among pigment‐binding complexes was investigated by inducing violaxanthin de‐epoxidation *in vitro* in isolated thylakoids from *C*.* vulgaris*, as reported in Fig. 3. Pigment‐binding complexes were then separated by native Deriphat‐PAGE electrophoresis (Figs S8–S10; Dreyfuss & Thornber, 1994), and zeaxanthin accumulation was investigated in eluted fractions by HPLC analysis. Only in the case of fractions corresponding to monomeric LHC, PSII core, and PSI complexes was a detectable amount of zeaxanthin found (Table S2). In the case of monomeric LHC, a substoichiometric amount of zeaxanthin was found. Considering the similar absorption spectrum of monomeric (fraction A1/B1) and trimeric LHC (fraction A3/B3), it is likely that the monomeric LHC fractions were mainly composed of monomeric LHCII, where zeaxanthin has been reported to be accumulated in the highly unstable external V1 site (Caffarri *et al*., 2001; Xu *et al*., 2015). The finding of zeaxanthin in the PSII core is instead related to a contamination from residual bound antenna complexes, since xanthophyll binding sites are absent in the PSII core. By cotrast, in the case of PSI complex, almost two zeaxanthin molecules per PSI were detected, with a DI of 0.34. The accumulation of zeaxanthin in monomeric LHC complexes did not significantly affect their absorption or 77 K fluorescence spectra, whereas a red shift in fluorescence spectra was evident in the case of PSI complex (Fig. S6). These results agree with those previously reported in the case of zeaxanthin‐binding PSI isolated from a *A*.* thaliana npq2* mutant (Ballottari *et al*., 2014), a mutant constitutively accumulating this xanthophyll (Niyogi *et al*., 1998). The quenching properties of zeaxanthin bound to monomeric LHC or to PSI complexes were then investigated by time‐resolved fluorescence (Figs 6, S11–S13). In the case of monomeric LHC, the fluorescence decay kinetics were not significantly changed by the presence of zeaxanthin. In order to investigate a possible quenching effect of zeaxanthin bound to trimeric LHCII, a milder solubilization of thylakoids was performed and pigment‐binding complexes were extracted by ultracentrifugation in a sucrose gradient, a procedure previously reported to partially maintain the occupancy of the V1 site (Caffarri *et al*., 2001). As reported in Table S2, the LHCII trimers obtained by this protocol were characterized by an increased xanthophyll content with a DI of 0.53 in the case of a complex isolated from de‐epoxidated thylakoids (LHCII‐Zea). However, no significant zeaxanthin‐dependent quenching was detectable, comparing LHCII with or without zeaxanthin in the V1 site (Fig. S11). By contrast, in the case of the PSI complex, a faster decay was evident in the zeaxanthin binding sample. In particular, an average fluorescence lifetime of 72 ± 4 ps was calculated in the case of the PSI complex in the absence of zeaxanthin, which decreased to 49 ± 7 ps in zeaxanthin binding PSI (Table S3). The *c*. 70 ps average fluorescence lifetime in zeaxanthin‐free PSI is consistent with the previous data reported in the case of *C*.* reinhardtii* (Le Quiniou *et al*., 2015), whereas the 30% reduction in average fluorescence lifetime observed in zeaxanthin binding PSI is consistent with the zeaxanthin‐dependent quenching previously observed in the case of zeaxanthin‐binding PSI from *A*.* thaliana* (Ballottari *et al*., 2014).

## Discussion

This work identifies a plant‐like VDE enzyme in *C*.* vulgaris* and relates its activity to the NPQ photoprotective mechanism of both PSI and PSII. *Chlorella vulgaris* is one of the leading microalgae at industrial level owing to its high growth rate and resistance to biotic and abiotic stresses (Liang *et al*., 2009; Du *et al*., 2012; Zuliani *et al*., 2016; Sarayloo *et al*., 2017; Cecchin *et al*., 2019). Recently, further increase of productivity in this microalga has been reported by increasing its resistance to photooxidative stress (Dall'Osto *et al*., 2019). Photoprotective mechanisms such as NPQ have been identified as key targets for biotechnological manipulation of photosynthetic organisms, assuring, on the one hand, enough photoprotection and, on the other hand, higher photosynthetic efficiency (Kromdijk *et al*., 2016). Zeaxanthin has been associated with different photoprotective functions, from singlet and triplet Chl excited‐state quenching to ROS scavenging, in both higher plants and green algae (Havaux & Niyogi, 1999; Baroli & Niyogi, 2000; Dall'Osto *et al*., 2012). However, the identification of CVDE, the gene product responsible for violaxanthin de‐epoxidation in *C*.* reinhardtii*, revealed a divergence in the evolution in the green lineage of the enzyme carrying the VDE catalytic activity, which is not homologous to the VDE of *A*.* thaliana*, but more similar to a lycopene cyclase (Li *et al*., 2016). Moreover, CVDE is in the stromal side of thylakoid membranes and it is not activated by lumen acidification (Li *et al*., 2016). In the case of Chlorophyta, a different distribution of VDE and CVDE enzymes could be found, as reported in Table S1, with both enzymes being found in the same genome only in the case of the Spaeropleales species investigated herein and in some Chlorellales, among which is *C*.* vulgaris*. However, these Trebouxiophyceae CVDE‐like genes appeared to be more phylogenetically distant compared with the other CVDEs (Fig. S1), and the catalytic function of the encoded genes should be further analysed in detail. The divergence between CVDE and VDE, despite a similar catalytic activity, demonstrates the plasticity of the carotenoid biosynthetic pathway, and the divergent evolution of the key enzyme involved is likely driven by specific functions and interactions with the environment. In this work, a VDE catalytic activity inducible at low pH was found in *C*.* vulgaris*, which allowed, using genome mining, identication of a conserved plant‐like VDE enzyme in this member of the Chlorophyta group. Phylogenetic analysis reveals, indeed, that VDE sequences are widely distributed in higher and lower plants. In unicellular algae, on the other hand, plant‐like VDE sequences could be found only in some species, among which are some green algae, diatoms, Haptophyta, Ochrophyta, and photosynthetic Alveolata such as *C*.* velia*. Other enzymes with lipocalin domains related to VDE can then be identified as VDR and VDL enzymes, but with possible different functions than violaxanthin de‐epoxidation. In the case of *C*.* vulgaris*, the VDE protein encoded by its genome displayed a high level of identity with the VDE of *A*.* thaliana* (Fig. 1). Only in the case of residues involved in the protein sensitivity to pH was a partial conservation found in the VDE enzyme from *C*.* vulgaris* compared with the VDE proteins from higher plants. Accordingly, the pH‐dependent activation of *A*.* thaliana* and *C*.* vulgaris* VDE is different: despite a similar optimum at pH 5.1, the catalytic activity of the latter at higher pH is dramatically reduced compared with the former. This might be a consequence of the lower number of protonatable residues found in the lipocalin‐like domain, considering a possible cooperative regulation of pH sensing in VDE given by the different protonatable residues (Fig. 1). The activity of *C*.* vulgaris* VDE was completely inhibited by DTT *in vitro*, a condition where CVDE in *C*.* reinhardtii* was proven not to be active. Similarly, almost complete inhibition of violaxanthin de‐epoxidation could be observed *in vivo* in the presence of DTT (Fig. S4); considering the insensitivity of CVDE to DTT (Li *et al*., 2016), its possible role in the xanthophyll cycle activation in *C*.* vulgaris* appears to be minor.

In higher plants, the fastest component of the NPQ mechanism, qE, depends on the interaction of an LHC‐like protein called PSBS with other LHC proteins (Li *et al*., 2000, 2002; Fan *et al*., 2015). Xanthophyll cycle activation has an important, though not crucial, role in higher plants in the induction of NPQ, as observed in the *npq1* and *npq2* mutants of *A*.* thaliana*, lacking VDE and zeaxanthin epoxidase: *npq2* showed faster NPQ onset kinetics, whereas *npq1* was characterized by reduced, but not zeroed, NPQ phenotypes compared with WT (Niyogi *et al*., 1998) (Fig. S14). By contrast, in the case of *Physcomitrella patens*, VDE activity has been reported to be essential for NPQ induction (Pinnola *et al*., 2013). In microalgae, the role of de‐epoxidized xanthophylls in the NPQ process is highly species specific (Lavaud *et al*., 2004, 2012; Schumann *et al*., 2007; Quaas *et al*., 2015; Park *et al*., 2019). In *C*.* reinhardtii*, mutants that are unable to accumulate zeaxanthin show an induction of NPQ similar to the WT (Bonente *et al*., 2011; Girolomoni *et al*., 2019), thus demonstrating that zeaxanthin does not have a specific role in NPQ in that organism. In *Phaeodactylum triconornutum*, strains with a reduced level of diatoxanthin display lower induction of NPQ (Lavaud *et al*., 2012). The role of zeaxanthin was also studied in the stramenopile *Phaeomonas* sp., for which the NPQ level is correlated with its accumulation and is already active in the dark (Berne *et al*., 2018), whereas a mutant on VDE enzyme in *Nannochloropsis oceanica* resulted in impaired NPQ induction (Park *et al*., 2019). In this work, a reduced NPQ phenotype was evident when the VDE enzyme was partially inhibited, demonstrating a role of zeaxanthin in NPQ induction in *C*.* vulgaris*. This result is in line with previous findings, where NPQ amplitude and/or kinetics were affected in *C*.* vulgaris* upon treatment with DTT (Goss *et al*., 2006; Quaas *et al*., 2015). Moreover, an exponential correlation between the induction of NPQ and zeaxanthin accumulation was found, suggesting that additional components are contributing to NPQ induction, especially at higher actinic light intensities. These could be LHC‐like proteins involved in quenching, such as PSBS and light‐harvesting complex stress‐related (LHCSR), recently reported also in the case of *C*.* vulgaris* (Cecchin *et al*., 2019). Alternatively, other LHCII proteins that present protonatable sites (Walters *et al*., 1996; Li *et al*., 2004; Liguori *et al*., 2013; Ballottari *et al*., 2016) could be responsible for the modulation of the extent of NPQ at different actinic light intensities, independently from the contribution of zeaxanthin. In *C*.* vulgaris*, zeaxanthin was also found to be involved in PSI quenching (Figs 5, 6) in both light‐dependent (Fig. 5) and light‐independent mechanisms (Fig. 6). Light‐dependent PSI quenching has been previously reported to be focused on its associated LHC antenna complexes and, in *C*.* reinhardtii*, to be modulated by LHCSR subunits by a zeaxanthin‐independent mechanism (Kosuge *et al*., 2018; Girolomoni *et al*., 2019). By contrast, in the case of *C*.* vulgaris*, inhibition of VDE activity caused a strong reduction of PSI light‐dependent quenching, suggesting a key role of zeaxanthin in this photoprotective mechanism, which from the data available, however, cannot be assessed as LHCSR dependent or independent (Fig. 5). In the case of PSI, a light‐independent quenching was also observed mediated by zeaxanthin when zeaxanthin‐binding complexes were isolated from thylakoid membrane. This photoprotective mechanism is consistent with previous findings in PSI complexes isolated from *A*.* thaliana* mutant that constitutively accumulated zeaxanthin, the mutant *npq2* (Niyogi *et al*., 1998; Ballottari *et al*., 2014). In PSI complexes isolated from *npq2*, all the violaxanthin binding sites were occupied by zeaxanthin, whereas in the zeaxanthin binding complex isolated from *C*.* vulgaris* we observed a DI of 0.34, with almost two zeaxanthin molecules per PSI complex that are likely bound to the external LHC antenna complexes (Wehner *et al*., 2004). When the PSI complex was isolated from *A*.* thaliana* with a similar DI and zeaxanthin : PSI stoichiometry, no evident effect of zeaxanthin in PSI quenching was observed (Tian *et al*., 2017), suggesting a much higher influence of zeaxanthin in PSI quenching in *C*.* vulgaris* than in *A*.* thaliana*. All these findings thus demonstrate the role of zeaxanthin in NPQ and excitation energy quenching in both PSII and PSI. Nevertheless, knockout mutants on VDE, LHCSR, and/or PSBS subunits in *C*.* vulgaris* are required to fully understand the relative role of these subunits in safe thermal dissipation of excitation energy absorbed by PSI and/or PSII.

It is interesting to note that the similar relationship between NPQ and zeaxanthin and the similar characteristics of the VDE enzyme in higher plants and *C*.* vulgaris* may be correlated with the capacity of these algae to form biofilms on land surface (Leliaert *et al*., 2012). Forming a living biofilm indeed increases the risk of being exposed to rapid light changes, as in the case of lower or higher plants (Quaas *et al*., 2015). However, it is important to note that other species known to live even in soils, such as *C*.* reinhardtii*, have a CVDE‐dependent xanthophyll cycle and the VDE enzyme can also be found in green algae living in planktonic form, including species such as *Ostreoccus* or *Micromonas*, preventing a direct correlation between VDE evolution and the biofilm vs planktonic living form of Chlorophyta. However, considering the minor role of zeaxanthin in NPQ in *C*.* reinhardtii* compared with the key role of zeaxanthin in *C*.* vulgaris* photoprotection, it is possible to speculate that the latter evolved photoprotective mechanisms that have proved to be successful in the case of higher plants, in which zeaxanthin plays a central role. According to this evolutionary perspective, it was reported that mosses, the earliest photosynthetic organisms conquering the land, present an NPQ mechanism fully dependent on zeaxanthin accumulation (Pinnola *et al*., 2013). Then, evolution of higher plants further tuned the dependency of photoprotection mechanisms toward the xanthophyll cycle, which maintained a central role in photoprotection (Fig. S10) (Havaux & Niyogi, 1999).

## Author contributions

MB conceived the study and designed and supervised the experiments. LG, FB and SC performed or contributed to all the experiments reported herein. GC and CD’A designed, coordinated, and supervised the fluorescence lifetime measurements. FP, CD’A and GdlCV performed fluorescence lifetime measurements. MB wrote the manuscript with the contribution of LG, FB, SC, CD’A and GC All the authors discussed the results, contributed to data interpretation, and commented on the manuscript. LG and FB contributed equally to this work.

## Supporting information


**Fig. S1** Phylogenetic tree of CVDE and CruP proteins.
**Fig. S2** HPLC chromatograms of pigments extracted from thylakoids after *in vitro* de‐epoxidation
**Fig. S3** Zeaxanthin content in thylakoids after *in vitro* de‐epoxidation
**Fig. S4** Effect of DTT on *Chlorella vulgaris* xanthophyll cycle and PSII fluorescence quantum yield.
**Fig. S5** Effect of DTT on NPQ kinetics of *Chlamydomonas reinhardtii*.
**Fig. S6** Pigment analysis on cells treated at different light intensities.
**Fig. S7** NPQ kinetics at different irradiances and their correlation with xanthophyll cycle.
**Fig. S8** Native Deriphat‐PAGE loaded with solubilized *Chlorella vulgaris* thylakoid membranes before and after in vitro de‐epoxidation reaction.
**Fig. S9** 77K fluorescence emission of bands isolated from Deriphat‐PAGE gel.
**Fig. S10** Absorption spectrum of bands isolated from Deriphat‐PAGE gel.
**Fig. S11** Fluorescence decay kinetics of trimeric LHCII complexes isolated from sucrose gradients.
**Fig. S12** Fluorescence decay kinetics of PSI complexes in the ns timescale.
**Fig. S13** Fluorescence decay kinetics of A2/B2, A3/B3, A4/B4 and A5/B5 fractions isolated from Deripaht‐PAGE gel.
**Fig. S14** NPQ kinetics of *Arabidopsis thaliana* in presence or absence of zeaxanthin.
**Methods S1** Primers and VDE sequences.
**Table S1** Identification of VDE, CVDE or CruP in different Chlorophyta.
**Table S2** HPLC analysis of monomeric LHC and PSI complexes isolated from control or *in vitro* de‐epoxidated thylakoids.
**Table S3** Fluorescence lifetimes of isolated pigments binding complexes.
Please note: Wiley‐Blackwell are not responsible for the content or functionality of any supporting information supplied by the authors. Any queries (other than missing material) should be directed to the *New Phytologist* Central Office.
