# Supplementary material for: Evolutionary divergence of photoprotection in the green algal lineage: a plant‐like violaxanthin de‐epoxidase enzyme activates the xanthophyll cycle in the green alga Chlorella vulgaris modulating photoprotection
Source: New Phytol. 2020 Jun 16;228(1):136–50. doi: 10.1111/nph.16674 (PMC7539987; doi:10.1111/nph.16674)
Supplement: Supplementary file 1 — Fig. S1 Phylogenetic tree of CVDE and CruP proteins. Fig. S2 HPLC chromatograms of pigments extracted from thylakoids after in vitro de‐epoxidation Fig. S3 Zeaxanthin content in thylakoids after in vitro de‐epoxidation Fig. S4 Effect of DTT on Chlorella vulgaris xanthophyll cycle and PSII fluorescence quantum yield. Fig. S5 Effect of DTT on NPQ kinetics of Chlamydomonas reinhardtii. Fig. S6 Pigment analysis on cells treated at different light intensities. Fig. S7 NPQ kinetics at different irradiances and their correlation with xanthophyll cycle. Fig. S8 Native Deriphat‐PAGE loaded with solubilized Chlorella vulgaris thylakoid membranes before and after in vitro de‐epoxidation reaction. Fig. S9 77K fluorescence emission of bands isolated from Deriphat‐PAGE gel. Fig. S10 Absorption spectrum of bands isolated from Deriphat‐PAGE gel. Fig. S11 Fluorescence decay kinetics of trimeric LHCII complexes isolated from sucrose gradients. Fig. S12 Fluorescence decay kinetics of PSI complexes in the ns timescale. Fig. S13 Fluorescence decay kinetics of A2/B2, A3/B3, A4/B4 and A5/B5 fractions isolated from Deripaht‐PAGE gel. Fig. S14 NPQ kinetics of Arabidopsis thaliana in presence or absence of zeaxanthin. Methods S1 Primers and VDE sequences. Table S1 Identification of VDE, CVDE or CruP in different Chlorophyta. Table S2 HPLC analysis of monomeric LHC and PSI complexes isolated from control or in vitro de‐epoxidated thylakoids. Table S3 Fluorescence lifetimes of isolated pigments binding complexes. Please note: Wiley‐Blackwell are not responsible for the content or functionality of any supporting information supplied by the authors. Any queries (other than missing material) should be directed to the New Phytologist Central Office. [file NPH-228-136-s001.pdf]

New Phytologist

***New Phytologist* Supporting Information**

Article title: **Evolutionary divergence of photoprotection in the green algal lineage: a plant-like Violaxanthin De-Epoxidase enzyme activates the xanthophyll cycle in the green alga Chlorella vulgaris modulating photoprotection**

Authors: Laura Girolomoni[a1], Francesco Bellamoli[a1], Gabriel de la Cruz Valbuena[b], Federico Perozeni[a], Cosimo D'Andrea[b,c], Giulio Cerullo[b], Stefano Cazzaniga[a], Matteo Ballottari[a*]

Article acceptance date: 13 May 2020

The following Supporting Information is available for this article:

Fig. S1 Phylogenetic tree of CVDE and CruP proteins.

Fig. S2 HPLC chromatograms of pigments extracted from thylakoids after *in vitro* de-epoxidation

Fig. S3 Zeaxanthin content in thylakoids after *in vitro* de-epoxidation

Fig. S4 Effect of DTT on *Chlorella vulgaris* xanthophyll cycle and PSII fluorescence quantum yield.

Fig. S5 Effect of DTT on NPQ kinetics of *Chlamydomonas reinhardtii*.

Fig. S6 Pigment analysis on cells treated at different light intensities.

Fig. S7 NPQ kinetics at different irradiances and their correlation with xanthophyll cycle.

Fig. S8 Native Deriphat-PAGE loaded with solubilized *Chlorella vulgaris* thylakoid membranes before and after *in vitro* de-epoxidation reaction.

Fig. S9 77K fluorescence emission of bands isolated from Deriphat-PAGE gel.

Fig. S10 Absorption spectrum of bands isolated from Deriphat-PAGE gel.

Fig. S11 Fluorescence decay kinetics of trimeric LHCII complexes isolated from sucrose gradients.

Fig. S12 Fluorescence decay kinetics of PSI complexes in the ns timescale.

Fig. S13 Fluorescence decay kinetics of A2/B2, A3/B3, A4/B4 and A5/B5 fractions isolated from Deripaht-PAGE gel.

Fig. S14 NPQ kinetics of *Arabidopsis thaliana* in presence or absence of zeaxanthin.

Table S1 Identification of VDE, VDR, CVDE or CruP in different *Chlorophyta.*

Table S2 HPLC analysis of monomeric LHC and PSI complexes isolated from control or *in vitro* de-epoxidated thylakoids.

Table S3 Fluorescence lifetimes of isolated pigments binding complexes.

Methods S1 Primers and VDE sequences

Fig. S1 Phylogenetic tree of CVDE and CruP proteins. Phylogenetic tree was obtained by multiple alignment of protein sequences carrying a VDE lipocalin domain identified by InterPro (IPR010788). The units of branch length are residues substitution per site divided by the length of the sequence. Bootstrap values are reported in red

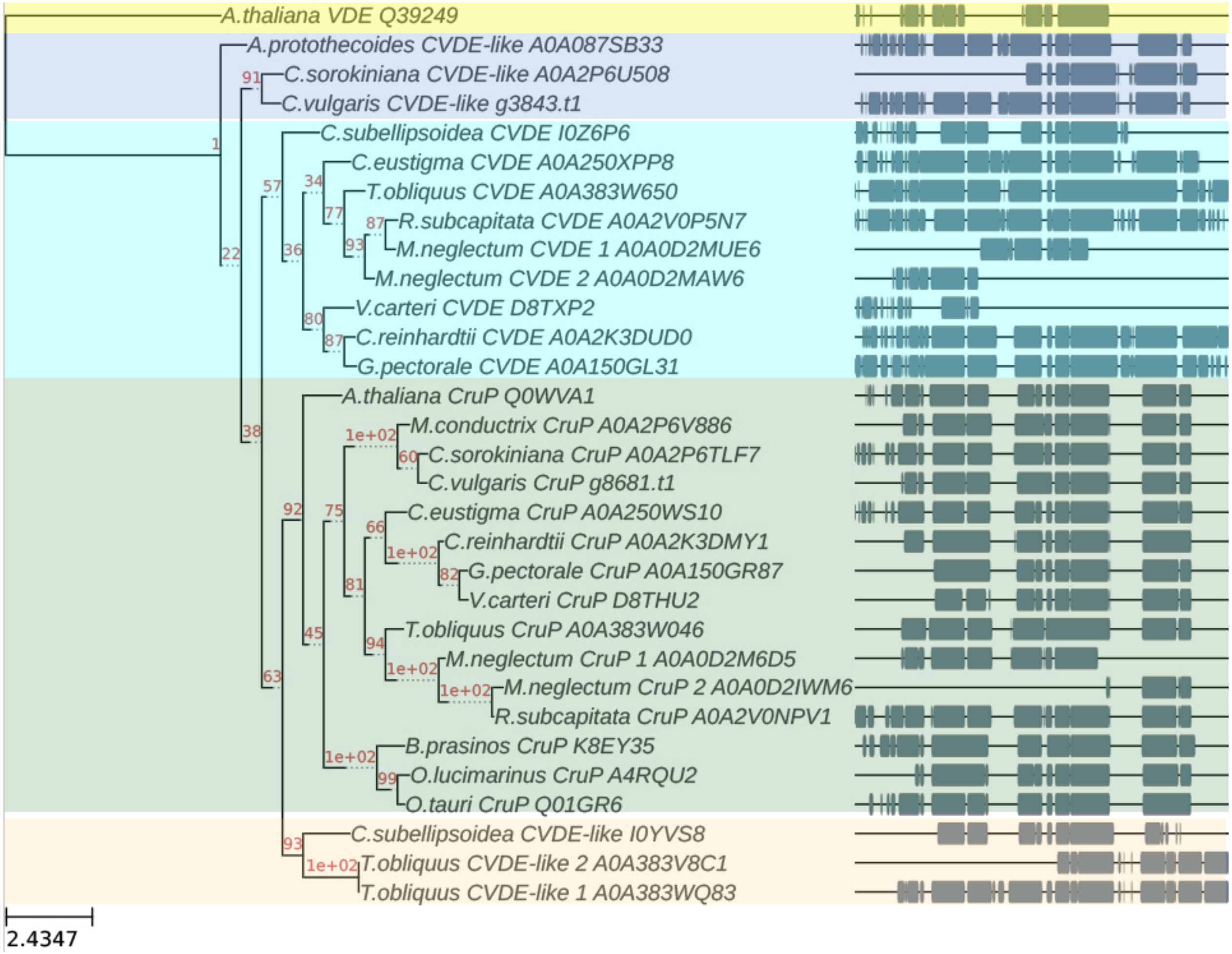

Fig. S2 HPLC chromatograms of pigments extracted from thyalkoids after *in vitro* de-epoxidation. Pigments were extracted in acetone 80% from thylakoids from spinach, *C. vulgaris* and *C. reinhardtii* after different times of incubation in de-epoxidation buffer. Chromatrogram were acquired measuring absorption at 400 nm.

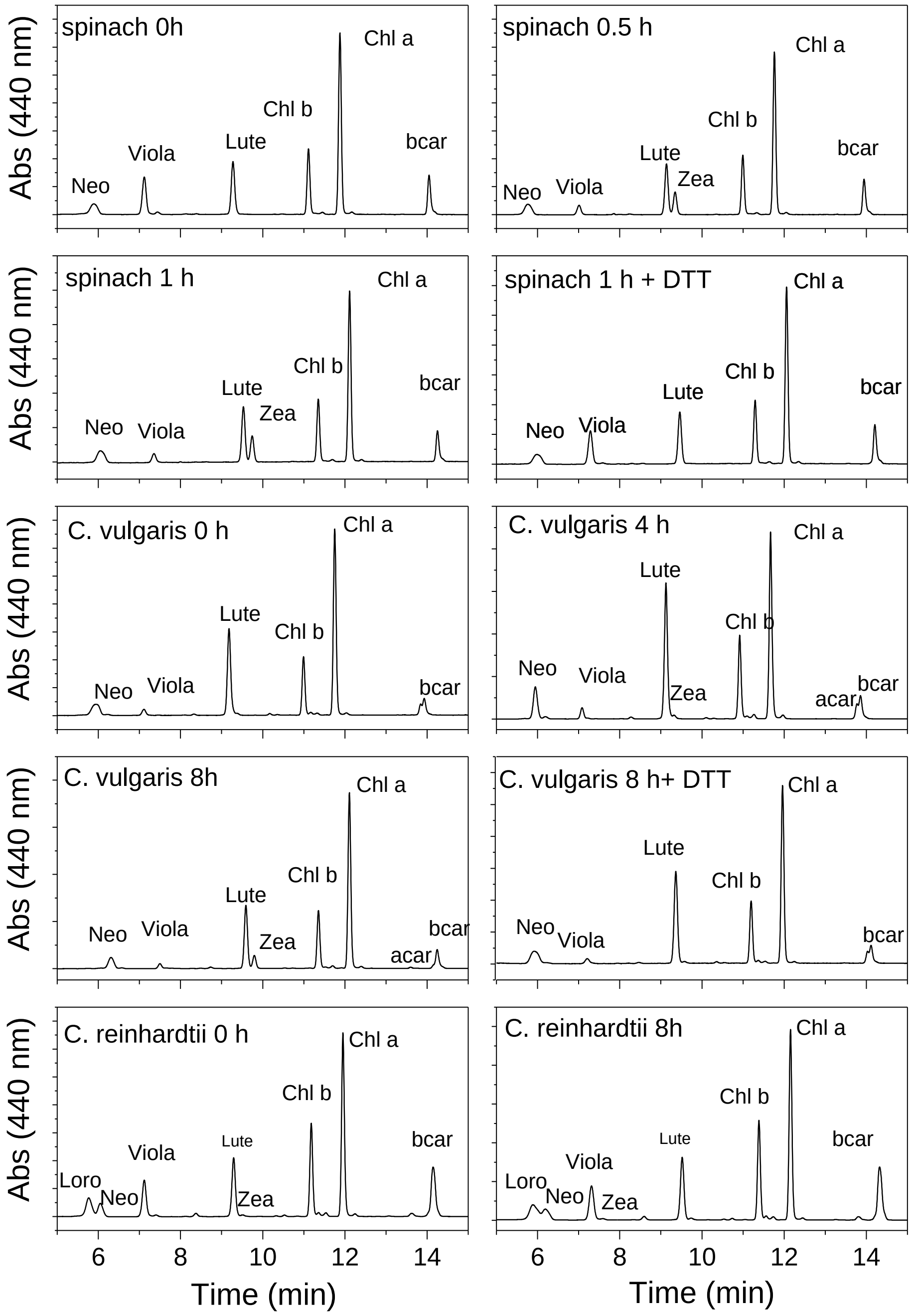

Fig. S3 Zeaxanthin and antheraxanthin content in thylakoids after *in vitro* de-epoxidation Zeaxanthin and antheraxanthin content normalized to 100 chlorophylls in thylakoids isolated from spinach and *C. vulgaris* (*C.v.*) before (t0) or after (DEP) 1 hours (spinach) or 8 hours (*C.v.*) at pH 5.1 in presence of ascorbate in order to induce violaxanthin de-epoxidation. Standard deviations are reported as error bars (n=3).

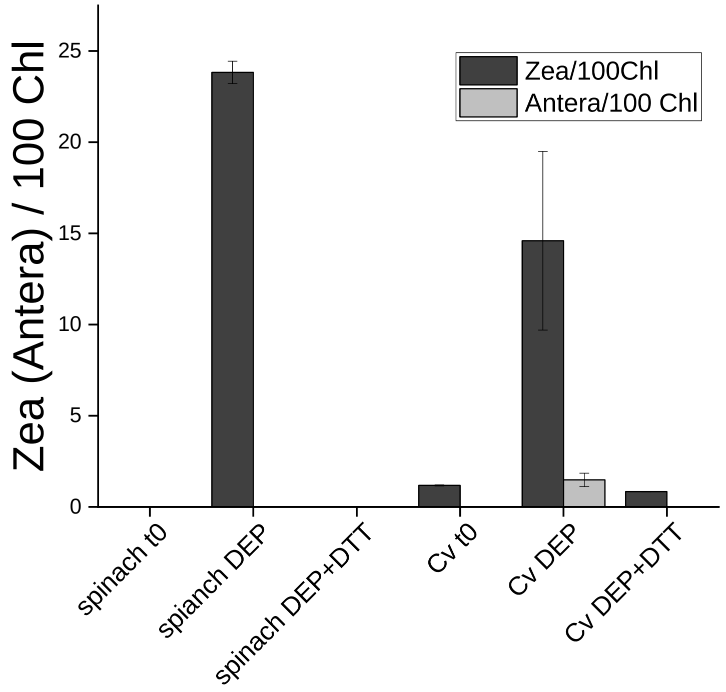

Fig. S4 Effect of DTT on *Chlorella vulgaris* xanthophyll cycle and PSII fluorescence quantum yield. De-epoxidation index (a) and zeaxanthin content (b), normalized to 100 chlorophylls of *C. vulgaris* exposed to 2000 µmol photons m^{-2} s^{-1} light for up to 40'. (c) PSII fluorescence quantum yield (Fv/Fm) of dark-adapted C. vulgaris cells in presence or absence of DTT. Standard deviations are reported as error bars (n=3).

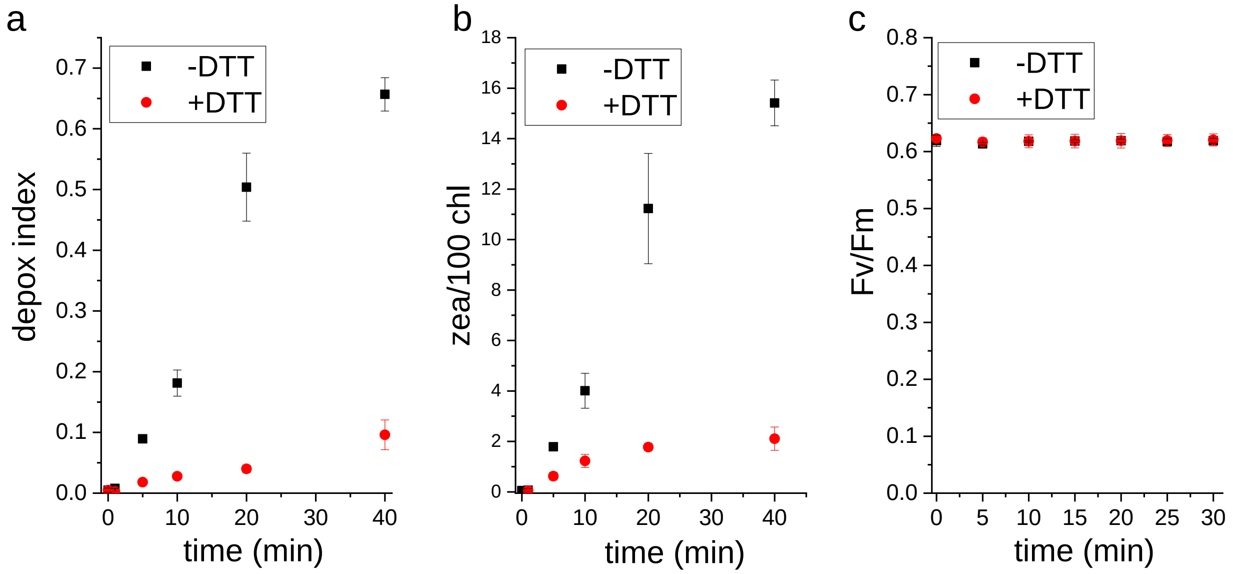

Fig. S5 Effect of DTT on NPQ kinetics of *Chlamydomonas reinhardtii*. Two consecutive cycles of NPQ induction in *C. reinhardtii* cells acclimated to high light are reported. High light acclimated cells were used for this experiment since in *C. reinhardtii* high light acclimation is required for NPQ induction. An actinic light of 2000 µmol photons m^{-2} s^{-1} and a saturating light of 4000 µmol photons m^{-2} s^{-1} was applied for this measurement. DTT was added to C. reinhardtii cells at a concentration of 1mM. Standard deviations are reported as error bars (n=3).

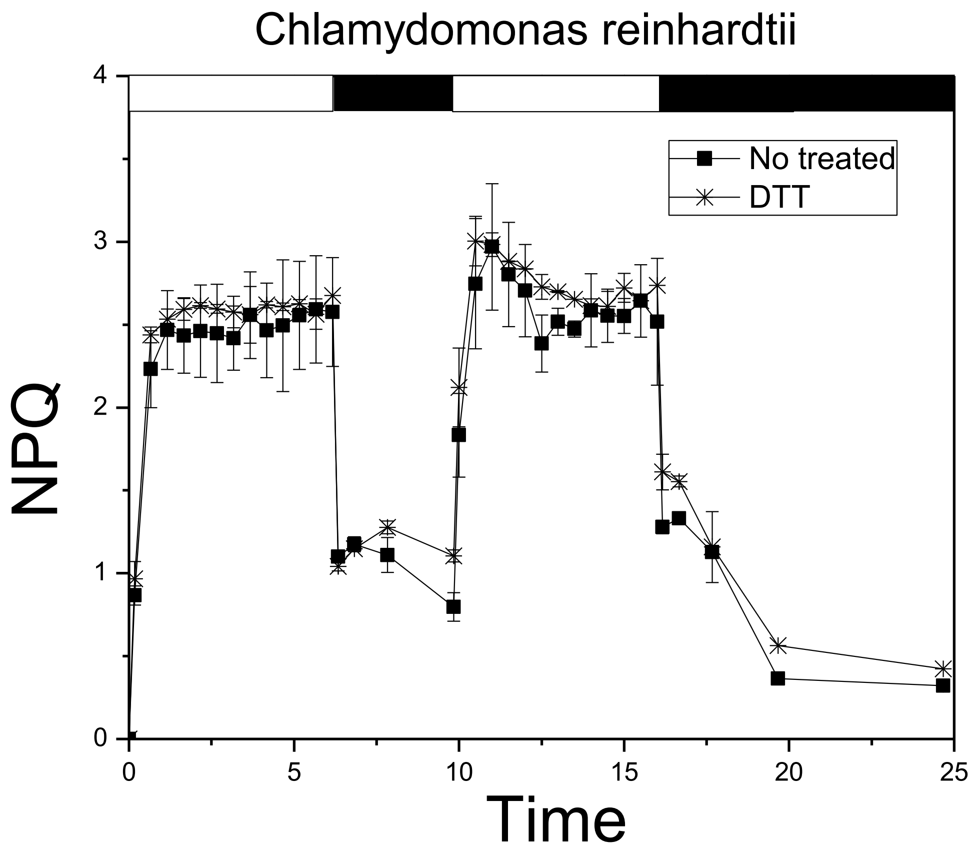

Fig. S6 Pigment analysis on cells treated at different light intensities. HPLC analysis of pigments extracted from *C. vulgaris* cells dark adapted (T0) or treated for 6 minutes with different actinic lights (200, 400, 800, 1200, 1800, 2000 and 2500 μmol photons m^{-2} s^{-1}). (**a**) Chlorophyll (Chl) a/b ratio and (**b**) Chl/carotenoid (Car) ratio, (**c**) the de-epoxidation index (D.I.) calculated a (zeaxanthin + 0.5x antearaxanthin)/(violaxanthin+zeaxanthin+antheraxanthin) and (**d**) Zeaxanthin (Zea) /Car ratio. Errors bars are reported as standard deviation (n=3).

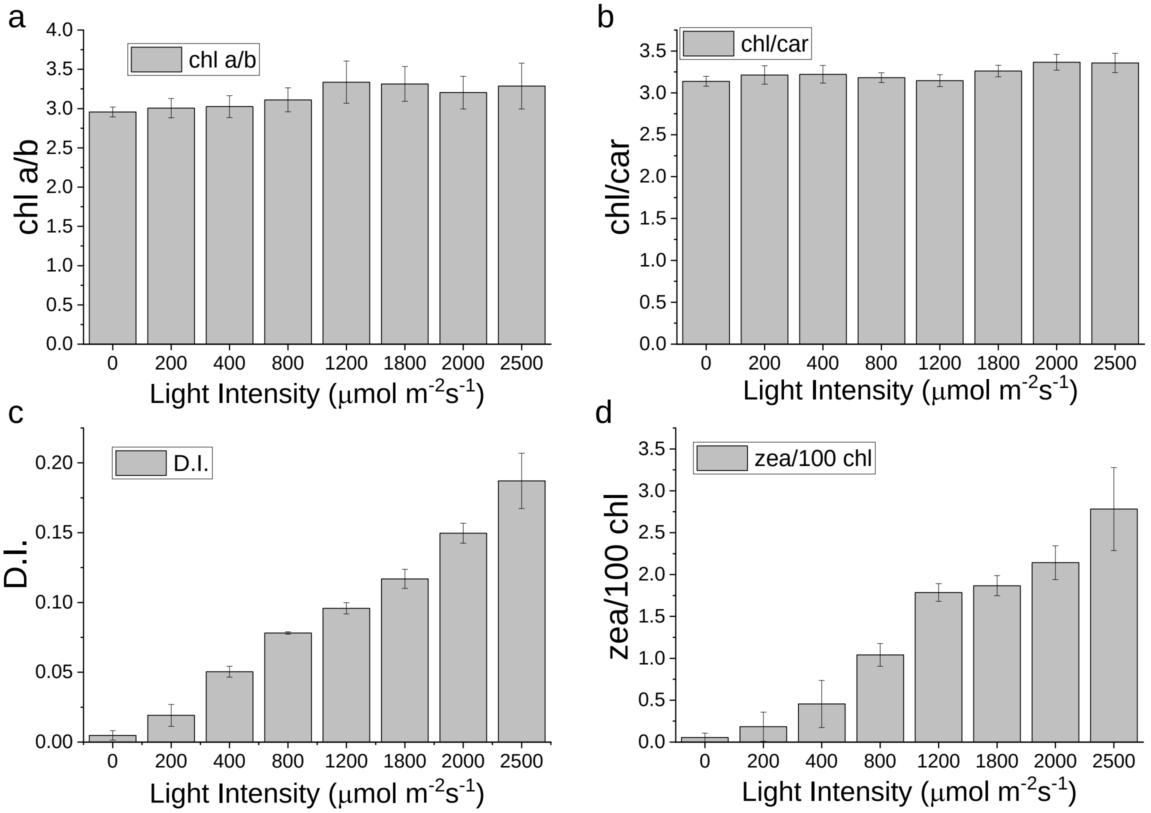

Fig. S7 NPQ kinetics at different irradiances and their correlation with xanthophyll cycle. (a): NPQ induction curves measured by using an actinic light of 200, 400, 800, 1200, 1800, 2000 and 2500 µmol photons m^{-2} s^{-1}. (b, c): correlation of de-epoxidation index (b) zeaxanthin content normalized to 100 chlorophylls (c) with NPQ , qE, and qI(qZ). NPQ was measured at the end of the actinic light exposure, while its components qE was measured as the NPQ component decaying in two minute in the dark, and qI(qZ), measured as the residual NPQ component after 9 minutes of dark relaxation. Standard deviations are reported as error bars (n=3). de-epoxidation index (D.I.), measured as reported in Figure S4, , at different light intensities. Panel C,D,E:

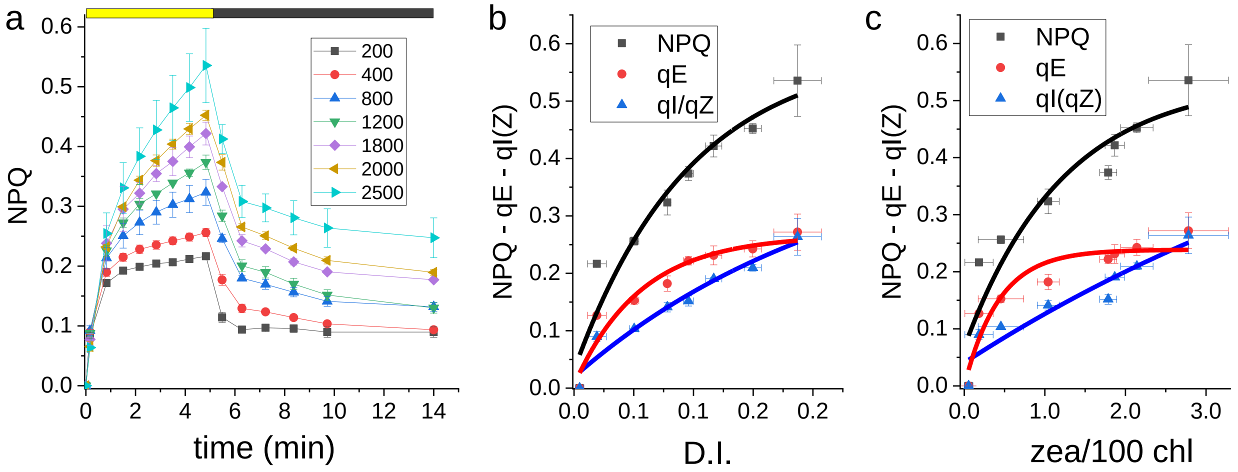

Fig. S8 Native Deriphat-PAGE loaded with solubilized *Chlorella vulgaris* thylakoid membranes before and after *in vitro* de-epoxidation reaction. Isolated thylakoid membranes were loaded before and after *in vitro* de-epoxidation reaction in order to induce zeaxanthin accumulation. Thylakoid membranes were de-epoxidated as reported in Figure 3. The different protein bands retrieved were eluted from the acrylamide matrix and numbered as reported in the figure (A: control, B: de-epoxidated thylakoids).

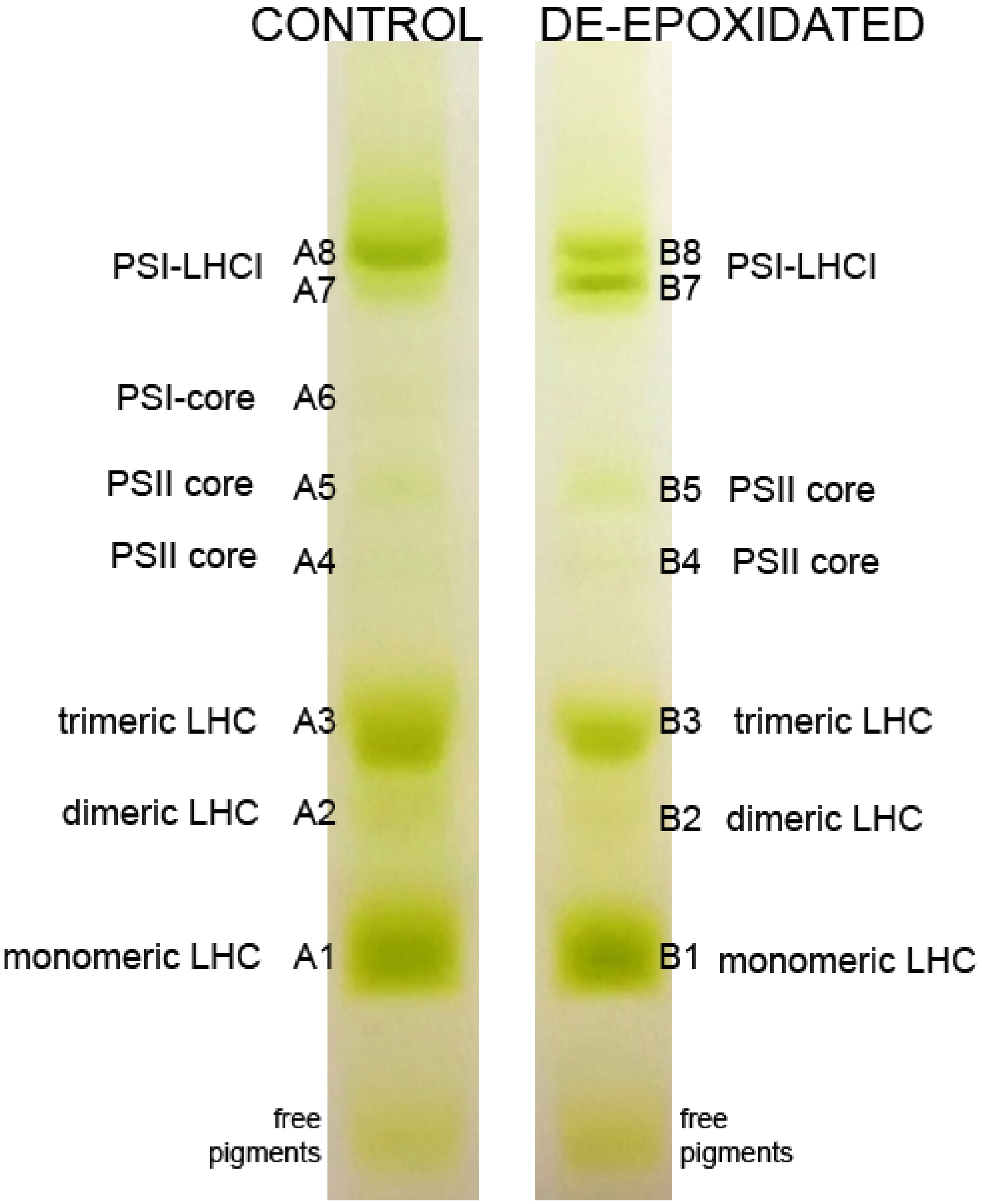

Fig. S9 77K fluorescence emission of bands isolated from Deriphat-PAGE gel. 77K fluorescence emission spectra were measured with excitation at 475 nm on bands cut from Deriphat-PAGE gel as reported in Figure S5 and eluted from acrylamide matrix in Hepes 20mM pH 7.5, sucrose 0.1M and 0.03% α-dodecylmaltosyde. Fractions A1-8 and B1-8 were isolated from control or de-epoxidated thylakoids, respectively. Fractions A1/B1, A2/B2, A3/B3 are respectively monomeric, dimeric and trimeric LHC; Fractions A4/B4 and A5/B5 are mainly composed by PSII core; A6 presents traces of PSI core, A7/B7 and A8/B8 are PSI complexes with different amount of LHCI, as previously reported in the case of *C. reinhardtii* (Le Quiniou *et al.*, 2015).

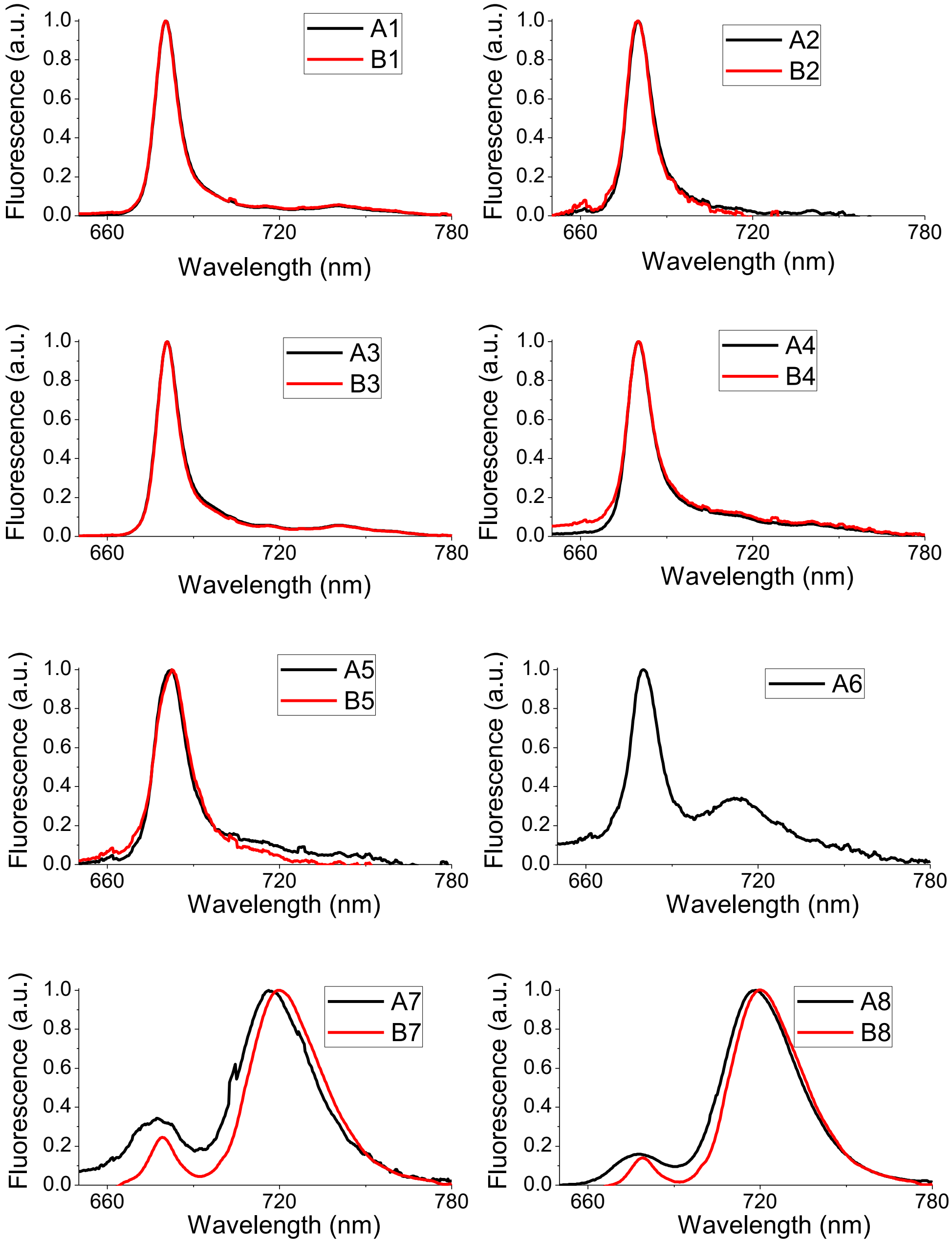

Fig. S10 Absorption spectrum of bands isolated from Deriphat-PAGE gel. Absorption spectra in the visible region spectra were measured on bands cut from Deriphat-PAGE gel as reported in Figure S5 and eluted from acrylamide matrix in Hepes 20mM pH 7.5, sucrose 0.1M and 0.03% α-dodecylmaltosyde. Fractions A1-8 and B1-8 were isolated from control or de-epoxidated thylakoids respectively. Fractions A1/B1, A2/B2, A3/B3 are respectively monomeric, dimeric and trimeric LHC; Fractions A4/B4 and A5/B5 are mainly composed by PSII core; A6 presents traces of PSI core, A7/B7 and A8/B8 are PSI complexes with different amount of LHCI, as previously reported in the case of *C. reinhardtii*.

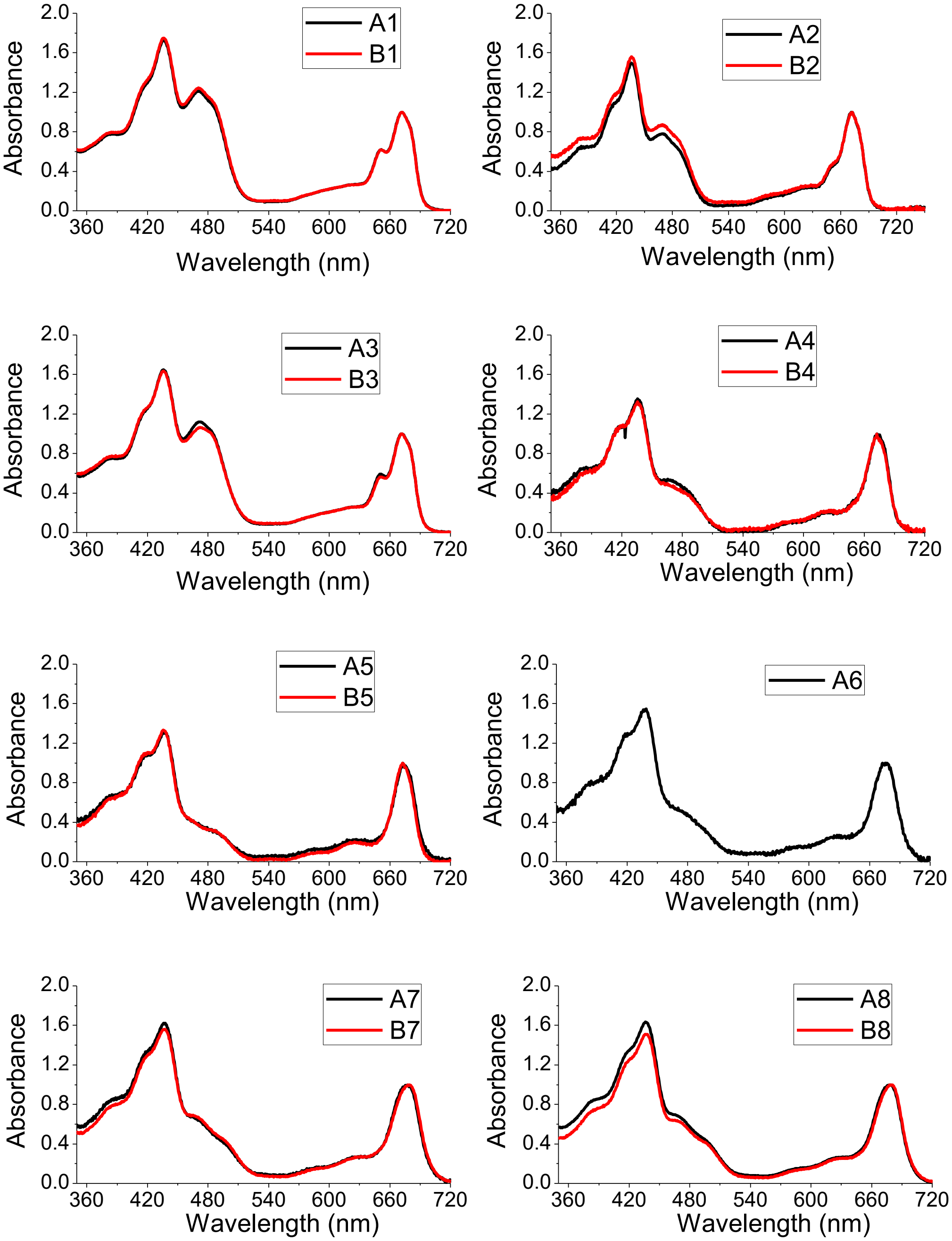

Fig. S11 Fluorescence decay kinetics of trimeric LHCII complexes isolated from sucrose gradients. Fluorescence decay kinetics of LHCII complexes isolated from sucrose gradient loaded with solubilized thylakoid membranes isolated from *C. vulgaris*. LHCII+Zea sample was purified from thylakoid membranes which were previously de-epoxidated, Fluorescence decay kinetics were measured by Streak camera CCD in TR4 mode (0-2000ps scale).

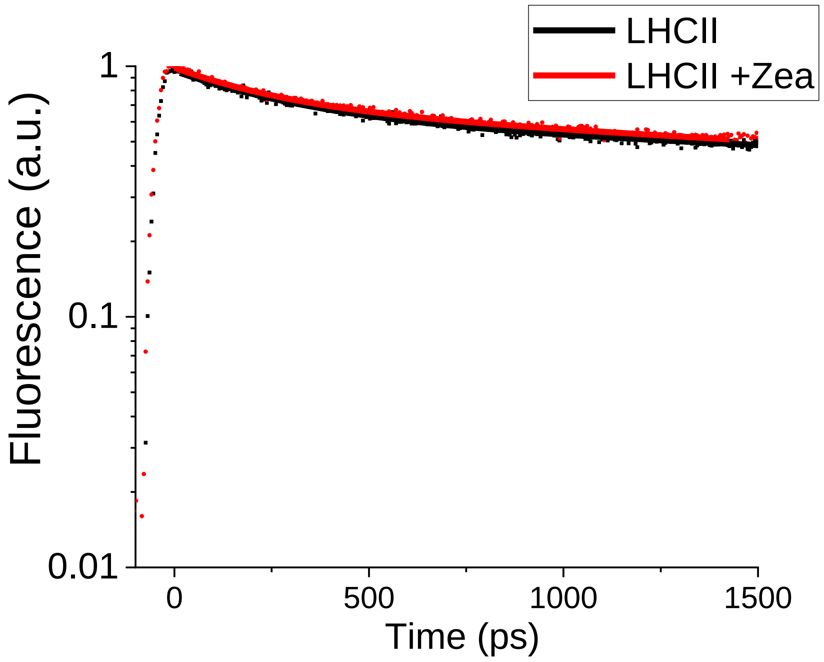

Fig. S12 Fluorescence decay kinetics of PSI complexes in the ns timescale. Fluorescence decay kinetics of PSI complexes isolated from thylakoid membranes before (A8 in Deriphat-PAGE reported in Figure S5) and after (B8 in Deriphat-PAGE reported in Figure S5) in vitro de-epoxidation were measured by Streak camera CCD in TR4 mode (0-2000ps scale).

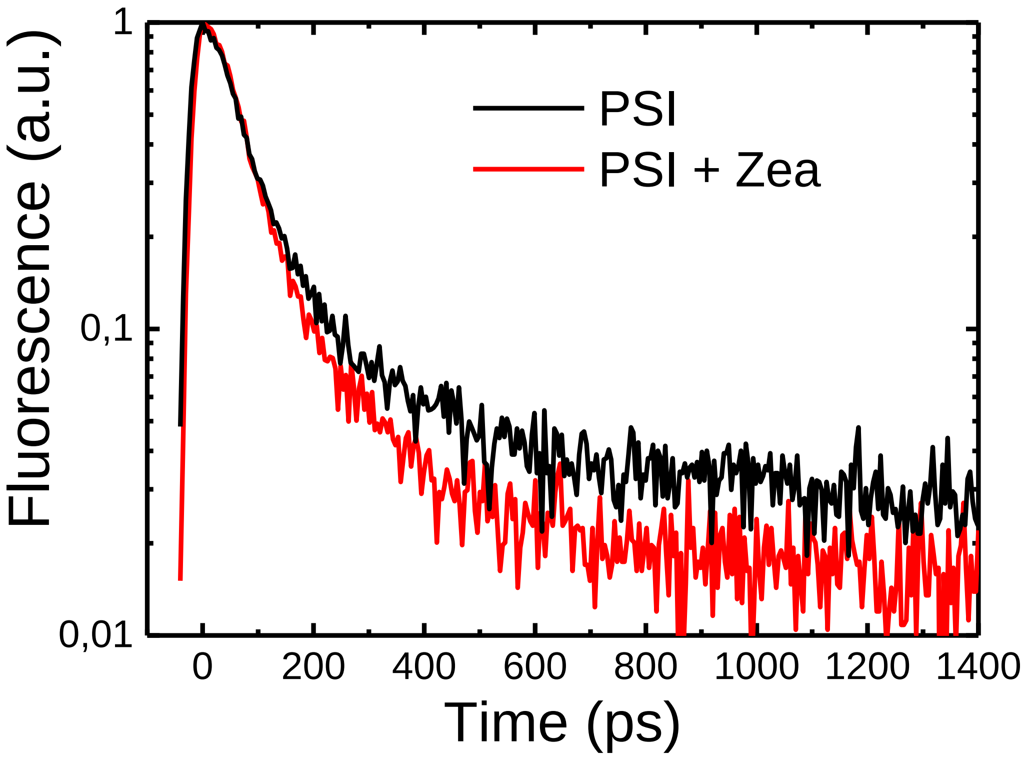

Fig. S13 Fluorescence decay kinetics of A2/B2, A3/B3, A4/B4 and A5/B5 fractions isolated from Deriphat-PAGE gel. Fluorescence decay kinetics of A2/B2, A3/B3, A4/B4 and A5/B5 fractions eluted from Deriphat-PAGE reported in Figure S5 were measured by TCSPC. No traces of zeaxanthin were found in B2, B3, B4 and B5 fractions despite their isolation from de-epoxidated thylakoids.

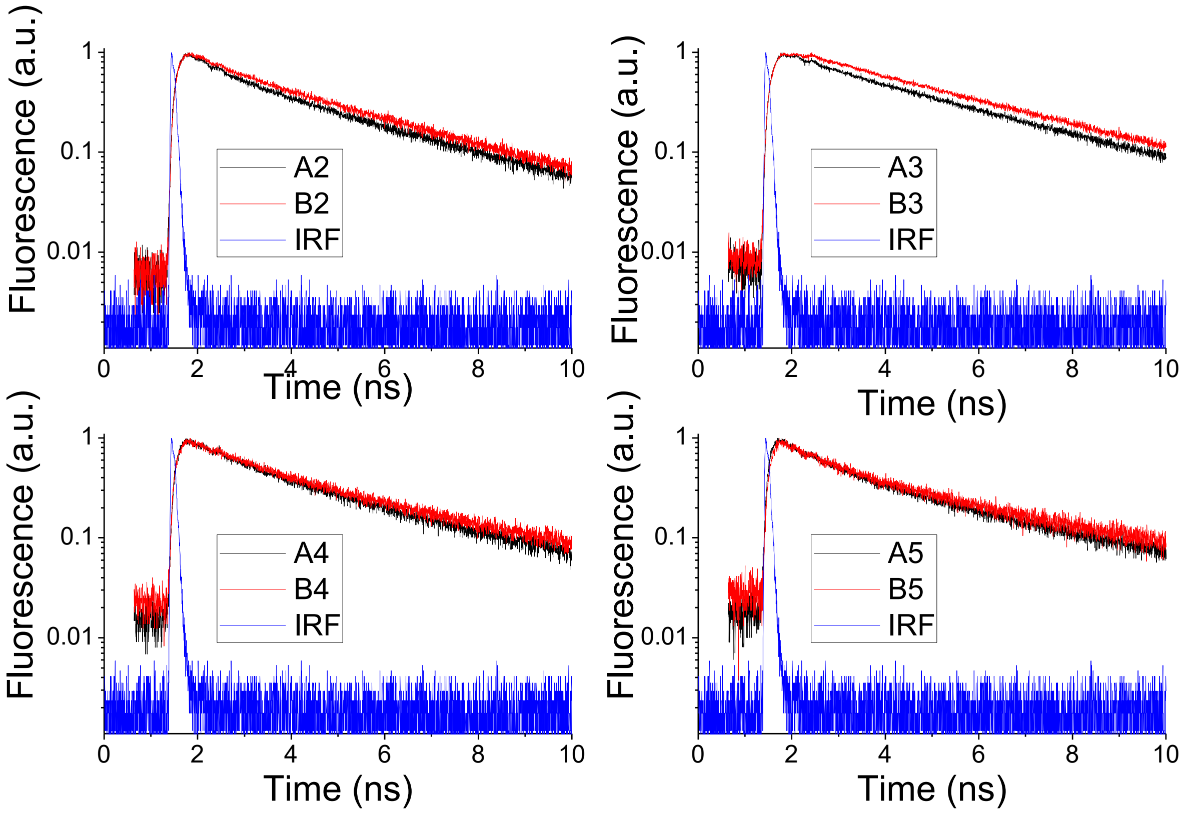

Fig. S14 NPQ kinetics of *Arabidopsis thaliana* in presence or absence of zeaxanthin. NPQ induction of *A. thaliana* WT, *npq1* and WT in presence of DTT are reported. *npq1* is mutant on *vde* gene in *A. thaliana.* An actinic light of 1200 µmol photons m^{-2} s^{-1} and a saturating light of 4000µmol photons m^{-2} s^{-1} was applied for this measurement. Standard deviations are reported as error bars (n=3).

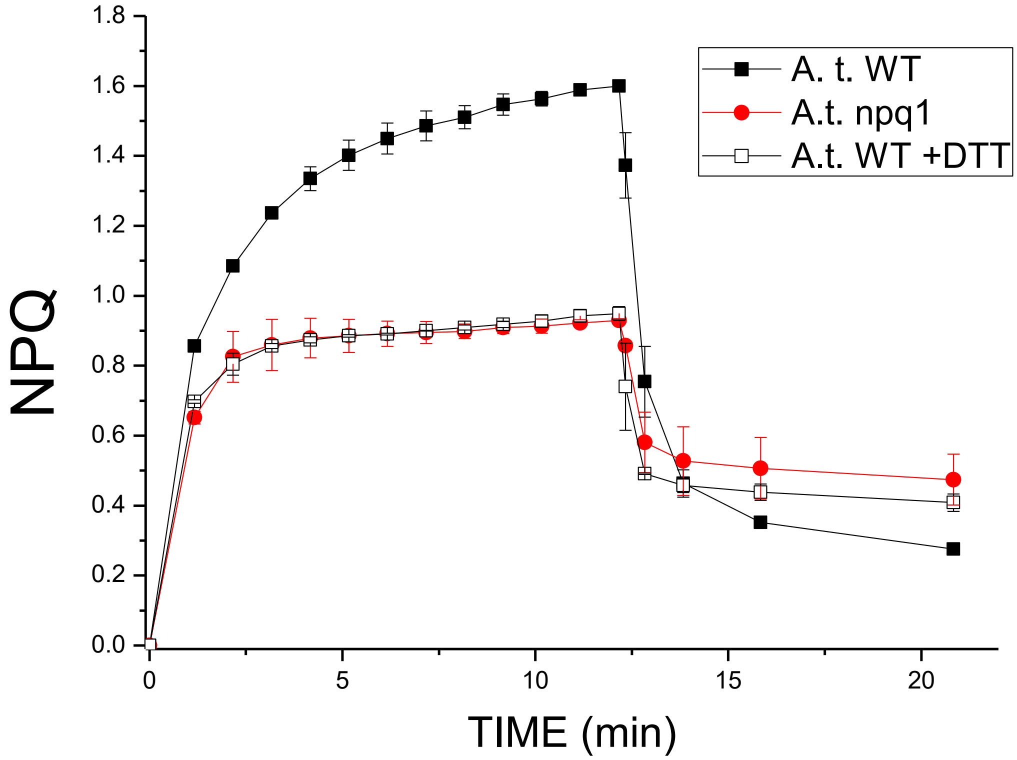

New Phytologist

Table S1 Identification of VDE, CVDE or CruP in different Chlorophyta. The presence of plant-like VDE and/or CVDE was in different green algae was performed by BLAST search using as queries the protein or cDNA sequence of A. thaliana VDE or C. reinhardtii CVDE.

Species	Class	Order	VDE	cVDE	CruP
Auxenochlorella protothecoides	*Trebouxiophyceae*	*Chlorellales*	X	X	
Chlorella sorokiniana	*Trebouxiophyceae*	*Chlorellales*		X	X
Chlorella variabilis	*Trebouxiophyceae*	*Chlorellales*	X		
Coccomyxa subellipsoidea (strain C-169)	*Trebouxiophyceae*	*Chlorellales*	X	X	X
Micractinium conductrix	*Trebouxiophyceae*	*Chlorellales*	X		X
Chlorella vulgaris	*Trebouxiophyceae*	*Chlorellales*	X	X	X
Monoraphidium neglectum	*Chlorophyceae*	*Sphaeropleales*	X	X	X
Raphidocelis subcapitata	*Chlorophyceae*	*Sphaeropleales*	X	X	X
Tetradesmus obliquus	*Chlorophyceae*	*Sphaeropleales*	X	X	X
Chlamydomonas eustigma	*Chlorophyceae*	*Volvocales*		X	X
Chlamydomonas reinhardtii	*Chlorophyceae*	*Volvocales*		X	X
Gonium pectorale	*Chlorophyceae*	*Volvocales*		X	X
Volvox carteri f. *nagariensis*	*Chlorophyceae*	*Volvocales*		X	X
Bathycoccus prasinos	*Mamiellophyceae*	*Mamiellales*	X		X
Micromonas commoda (strain RCC299 / NOUM17 / CCMP2709)	*Mamiellophyceae*	*Mamiellales*	X		
Micromonas pusilla (strain CCMP1545)	*Mamiellophyceae*	*Mamiellales*	X		
Ostreococcus lucimarinus (strain CCE9901)	*Mamiellophyceae*	*Mamiellales*	X		X
Ostreococcus tauri	*Mamiellophyceae*	*Mamiellales*	X		X

Table S2 HPLC analysis of isolated pigments binding complexes. Fractions isolated from Deriphat-PAGE loaded with solubilized control or in vitro de-epoxidated C. vulgaris thylakoids were analyzed by HPLC after pigment extraction by acetone 80%. LHCII isolated from sucrose gradient loaded with solubilized control or in vitro de-epoxidated C. vulgaris thylakoids are also reported indicated as LHCII (isolated from control thyalkoids) and LHCII+Zea (isolated from de-epoxidated thyalkoids. Chl: chlorophyll; Car: carotenoids; Neo: neoxanthin; viola: violaxanthin; lute: lutein; zea: zeaxanthin; b car: beta-carotene; n.d.: not detected. Relative errors are below 15% for each value (n=2).

	**Chl**	**Chl a/b**	**Car**	**neo**	**viola**	**anthera**	**lute**	**zea**	**b car**	**D.I.**
**A free pigments**	100	4.2	33.2	2.0	2.4	n.d.	24.6	n.d.	4.2	0.0
**A1**	12	1.9	3.2	0.8	0.2	n.d.	2.2	n.d.	0.1	0.0
**A2**	14	2.5	2.2	0.3	0.2	n.d.	1.3	n.d.	0.4	0.0
**A3**	14	1.5	2.4	0.7	0.1	n.d.	1.6	n.d.	n.d.	0.0
**A5**	100	13.6	11.5	n.d.	0.8	n.d.	4.3	n.d.	6.4	0.0
**A7**	170	11.0	27.5	n.d.	2.6	n.d.	8.1	n.d.	16.8	0.0
**A8**	170	6.7	35.7	0.7	4.9	n.d.	15.8	n.d.	14.3	0.0
	**Chl**	**Chl a/b**	**Car**	**neo**	**viola**	**anthera**	**lute**	**zea**	**b car**	**D.I.**
**B free pigments**	100	2.8	35.5	2.0	1.8	1.6	25.2	2.5	2.4	0.6
**B1**	12	1.9	3.5	1.0	0.1	n.d.	2.3	0.1	0.0	0.6
**B2**	14	2.2	3.1	0.7	0.1	n.d.	1.9	n.d.	0.3	0.0
**B3**	14	1.5	3.2	1.0	0.2	n.d.	2.1	n.d.	n.d.	0.0
**B5**	100	8.6	14.8	n.d.	0.4	n.d.	4.7	0.7	9.1	0.6
**B7**	170	5.4	29.0	n.d.	1.7	n.d.	10.2	1.2	15.9	0.4
**B8**	170	4.5	36.4	2.4	3.3	n.d.	14.9	1.7	14.0	0.3
	**Chl**	**Chl a/b**	**Car**	**neo**	**viola**	**anthera**	**lute**	**zea**	**b car**	**D.I.**
**LHCII**	14	2.3	3.3	0.6	0.1	n.d.	2.6	n.d.	0.1	0.0
**LHCII-ZEA**	14	2.2	3.4	0.5	0.1	0.1	2.5	0.1	0.2	0.5

Table S3 Fluorescence lifetimes of isolated pigments binding complexes. Fluorescence decay kinetics were fitted with 1, 2 or 3 exponential functions. The amplitudes (a1-3) and time constant (t1-3) retrieved are reported with the average fluorescence lifetime (τav) calculated as (a1*t1+a2*t2+a3*t3)/(a1+a2+a3). The values obtained for two different preparations for each sample are reported. The average value and standard deviations for the τav are also reported. n.d.: not determined by the fitting analysis.

	a1	τ1 (ps)	a2	τ2 (ps)	a3	τ3 (ps)	τav (ps)	a1	τ1 (ps)	a2	τ2 (ps)	a3	τ3 (ps)	τav (ps)	τav (ps) AVERAGE	st.dev.
**A1**	0.06	138	0.94	3070	n.d.	n.d.	2883	0.17	177	0.83	3500	n.d.	n.d.	2920	**2901**	**26**
**B1**	0.05	121	0.95	3260	n.d.	n.d.	3095	0.23	188	0.77	3851	n.d.	n.d.	2993	**3044**	**72**
**A2**	0.11	161	0.89	2820	n.d.	n.d.	2538	n.d.	n.d.	1.00	2685	n.d.	n.d.	2685	**2612**	**104**
**B2**	0.08	136	0.92	2950	n.d.	n.d.	2726	n.d.	n.d.	1.00	2886	n.d.	n.d.	2886	**2806**	**113**
**A3**	0.05	127	0.95	3220	n.d.	n.d.	3056	0.18	161	0.82	3592	n.d.	n.d.	2984	**3020**	**51**
**B3**	0.03	58	0.97	3280	n.d.	n.d.	3195	0.22	196	0.78	3697	n.d.	n.d.	2909	**3052**	**202**
**A4**	0.72	68	0.09	1110	0.18	3230	743	0.82	58	0.01	995	0.17	3546	653	**698**	**63**
**B4**	0.74	61	0.1	1290	0.17	3610	771	0.72	61	0.12	1250	0.16	3650	784	**777**	**9**
**A5**	0.77	75	0.1	1290	0.13	3610	640	0.85	69	0.03	1009	0.12	3750	544	**592**	**68**
**B5**	0.73	75	0.11	1130	0.16	3790	785	0.78	72	0.11	915	0.11	4326	645	**715**	**99**
**A8**	0.18	8	0.82	90	n.d.	n.d.	76	0.15	7	0.85	80	n.d.	n.d.	69	**72**	**4**
**B8**	0.34	12	0.66	77	n.d.	n.d.	54	0.39	11	0.61	67	n.d.	n.d.	45	**49**	**7**
**LHCII**	0.36	271	0.67	5147	n.d.	n.d.	3454	0.00	0	1.00	3450	n.d.	n.d.	3450	**3452**	**3**
**LHCII-Zea**	0.32	274	0.64	5140	n.d.	n.d.	3509	0.00	0	1.00	3383	n.d.	n.d.	3383	**3446**	**89**

Methods S1 Primers and VDE sequences

Primers sequences
Primers used for *vde* transcript amplification from cDNA

Forward sequence (5' -3')	Reverse sequence (5'-3')
ATATAAAGCTTATGGCAGCTGCAGCACGC	ATATACTCGAGATCCATGGGCATGATGACTG

VDE sequences
C. vulgaris VDE transcript and protein sequences. Chloroplast transit peptide predicted by ChloroP 1.1 is reported in bold, while the signal peptide predicted by TargetP 2.0 is underlined containing also a putative signal for protein import in thylakoid lumen.

>g7391.t1_protein
MQASRCTAAAVPAAPATNLPRCRRRVVRAAAARRPAASQQRDADRQQEAQQPQQLGLTPLQKVAT
GAAGLLASAVLLTAPGSALAADTAAVGTCLLQNCQAALAQCLTDVTCAENLVCLQLCNGRPDETECQIKC
GDKYSDKAVETFTACAVSEKKCVPQRIDEDAYPVPPDSALDNSFDLSNFQGRWYITAGLNPLFDTFDCQ
EHFFASPEPNKVFAKINWRIPMSDALTGDQDFIERSVMQKFVQEDPAKQPSVLVNKDNEFLNYQDTWYV
LAFKPDNYVFIYYRGQNDAWLGYGGATVYTRTSTLPREDIPELKAAAERAGLDWSKFTITNNSCPPHPPK
AALPEKLRVAAVRRTAQAEYELENDLRSFGRGFTVLEKDLSNKLRRTENTIVEDIKAVGQVEKKLEQTAGK
AEKLIEKEVEEVEAAAARMIRRFEAEAKMGPWINWIPKSWRPVIMPMD

>g7391.t1_transcript
ATGCAGGCCTCGAGGTGCACCGCAGCAGCCGTGCCAGCAGCCCCCGCCACAAACCTCCCGAGGT
GCCGCCGGCGTGTGGTGCGGGCAGCTGCAGCACGCCGCCCAGCCGCGTCTCAACAACAGCGCGA
TGCAGATAGGCAGCAGGAGGCACAACAGCCGCAGCAGCTGGGCCTGACCCCACTGCAGAAGGTGG
CAACTGGTGCGGCAGGCCTGCTAGCCTCTGCGGTCCTCCTCACGGCGCCTGGCTCAGCATTGGCG
GCAGACACTGCGGCTGTGGGCACGTGCCTGCTGCAAAACTGTCAAGCTGCGCTGGCCCAGTGCCT
CACAGACGTCACCTGCGCGGAGAACCTGGTGTGCCTGCAGCTGTGCAACGGCCGCCCAGACGAGA
CTGAGTGCCAGATCAAGTGTGGTGACAAGTATTCCGACAAGGCGGTGGAGACGTTCACTGCCTGCG
CAGTCAGCGAGAAGAAGTGTGTCCCGCAGCGAATTGACGAGGATGCCTACCCCGTGCCACCAGACA
GTGCACTTGACAACAGCTTCGATTTGTCGAACTTCCAGGGCCGCTGGTACATCACTGCTGGGCTAAA
CCCACTGTTCGACACATTCGACTGCCAGGAGCATTTCTTTGCCAGCCCGGAACCAAACAAGGTGTTT
GCCAAGATCAACTGGCGGATTCCCATGTCAGACGCTCTGACTGGGGATCAGGACTTCATTGAGCGG
TCTGTGATGCAGAAGTTTGTGCAGGAGGACCCTGCCAAGCAGCCTTCTGTCCTAGTGAACAAGGAC
AACGAATTTTTGAACTACCAAGACACTTGGTATGTGCTAGCTTTCAAGCCTGACAACTACGTCTTCAT
CTACTATCGAGGCCAGAATGATGCGTGGCTGGGCTACGGCGGCGCTACTGTTTACACACGCACCTC
GACCCTGCCTCGTGAGGACATTCCGGAGCTTAAGGCTGCAGCAGAGCGTGCGGGACTGGACTGGT
CCAAGTTCACCATCACCAACAACAGCTGCCCACCTCACCCGCCCAAGGCAGCCCTGCCCGAGAAGC
TGCGGGTGGCTGCGGTGCGCCGTACTGCCCAGGCTGAATATGAGCTTGAGAACGATCTGCGCTCCT
TTGGCCGAGGCTTCACCGTGCTAGAGAAGGATCTGTCAAATAAGCTGCGCCGCACTGAGAACACGA
TTGTGGAGGACATCAAGGCTGTGGGGCAGGTCGAAAAGAAGCTGGAGCAGACGGCGGGCAAGGCA
GAGAAGCTCATTGAGAAGGAGGTAGAAGAGGTGGAGGCAGCTGCGGCCCGCATGATTCGGCGCTT
TGAGGCAGAGGCAAAGATGGGACCCTGGATCAACTGGATTCCCAAGAGCTGGCGGCCAGTCATCAT
GCCCATGGATTGA

References

Le Quiniou C, Tian L, Drop B, Wientjes E, van Stokkum IH, van Oort B, Croce R. 2015. PSI-LHCI of Chlamydomonas reinhardtii: Increasing the absorption cross section without losing efficiency. *Biochim Biophys Acta* **1847**(4-5): 458-467.
